# Memory B Cell Repertoire of a Repeatedly Vaccinated Individual Includes Influenza Hemagglutinin-Specific Cross-Group Binders with Affinity Beyond A(H1N1)pdm09 and A(H3N2) Seasonal Strains

**DOI:** 10.3390/vaccines14090742

**Published:** 2026-08-27

**Authors:** Margarita Mishina, Christopher M. Horn, Zachary Ende, Vasiliy Mishin, Crystal Holiday, Sujatha Seenu, Jason R. Wilson, Weiping Cao, Mili Sheth, Dhwani Batra, Jessie Chang, Paul Carney, Min Z. Levine, James Stevens, Michael R. Weigand, Ian A. York, Shivaprakash Gangappa

**Affiliations:** 1 Influenza Division, Centers for Disease Control and Prevention, Atlanta, GA 30329, USA; oit8@cdc.gov (Z.E.); fwc7@cdc.gov (V.M.); isv4@cdc.gov (C.H.); iun1@cdc.gov (S.S.); vhd7@cdc.gov (J.R.W.); jgz9@cdc.gov (W.C.); vnj9@cdc.gov (J.C.); gul1@cdc.gov (P.C.); mwl2@cdc.gov (M.Z.L.); fwb4@cdc.gov (J.S.); ite1@cdc.gov (I.A.Y.); 2 Division of Core Laboratory Services & Response, Centers for Disease Control and Prevention, Atlanta, GA 30329, USA; kxc7@cdc.gov (C.M.H.); vgg1@cdc.gov (M.S.); yrh8@cdc.gov (M.R.W.); 3 Association of Public Health Laboratories, Bethesda, MD 20814, USA; 4 Division of Infectious Disease Readiness and Innovation, Centers for Disease Control and Prevention, Atlanta, GA 30329, USA; bun3@cdc.gov

**Keywords:** monoclonal antibody, Influenza, immune response, B cells, cross-reactive

## Abstract

**Background/Objectives**: Humans are primarily infected by two distinct seasonal influenza A virus (IAV) subtypes, A(H1N1)pdm09 and A(H3N2). Because IAV mutates rapidly, it continuously escapes existing protective antibodies. However, repeated exposure to these evolving strains increases the diversity of virus-specific memory B cell (MBC) population. To investigate the extent of this diversity and cross-reactivity of IAV- specific immunity in adults, and its possible impact on emerging strains, we analyzed the hemagglutinin (HA)-specific MBC repertoire of an adult frequently immunized with seasonal vaccines. **Methods**: High-throughput single-cell sequencing of HA probe-positive MBCs collected from an individual with repeated seasonal influenza vaccination was performed. Monoclonal antibodies (mAbs) from multicell clonotypes were tested for HA binding and influenza virus neutralization. **Results**: Two clonotypes were identified that bound to both A(H1) and A(H3) HAs from strains circulating before and after the season of blood collection. Both clonotypes neutralized A(H3N2) strains, while one also neutralized A(H1N1)pdm09 strains. Notably, these cross-group binders showed affinity for A(H5), A(H7) and A(H9) avian influenza HAs and neutralized bovine A(H5N1) IAV. **Conclusions**: Our findings suggest that seasonal vaccination can support pre-existing cross-reactive B-cell immunity in some individuals. This underscores the importance of studying influenza-specific MBC repertoires across diverse populations to better understand immune dynamics and improve vaccine design.

## 1. Introduction

Infections caused by influenza A viruses (IAVs) are major contributors to severe respiratory disease [1]. IAVs are divided into subtypes based on two surface proteins: HA, which is essential for viral entry to the host cell, and neuraminidase (NA), which facilitates release of newly formed virions from the cell [2]. Additionally, IAVs are classified into Group 1 and Group 2 based on HA subtype genetic and structural similarities [3]. IAVs evolve through two main mechanisms: mutations and segment reassortment, allowing the pathogen to evade immune detection and resist vaccines or treatments designed for earlier strains. A(H1N1)pdm09 and A(H3N2) IAVs currently circulate in humans seasonally and annual vaccination is recommended with updated vaccines to match the circulating strains. In addition to endemic human infections, IAVs present a persistent pandemic threat due to their genetic flexibility and ability to cross species barriers. In particular, the recent outbreaks of A(H5N1), A(H7N9) and A(H9N2) avian IAV subtypes have raised significant public health concerns [4].

B cells are essential for adaptive immunity and play a critical role in protection against influenza virus. To recognize a wide variety of pathogens, B cells collectively express an enormous number of unique surface immunoglobulins or B cell receptors (BCRs), forming the BCR repertoire [5]. The tremendous BCR variation is the result of the rearrangement of multiple variable (V), diversity (D) and joining (J) genes for heavy and light chains along with random deletion or insertion of nucleotides in segment junctions during development into mature naïve B cells, and somatic hypermutation for affinity maturation after antigen exposure. Both influenza infection and vaccination lead to B cell activation and subsequent differentiation into antibody-secreting cells (ASCs) and memory B cells (MBCs) for long-lasting protection. MBCs provide a rapid and robust secondary immune response, differentiating into ASCs much faster than naïve B cells after exposure to the same or similar antigens [6].

Influenza virus HA is the major viral surface protein. It is essential for initiating infection of the target cells and is the primary target for humoral immunity [7]. HA consists of a head domain, housing the receptor binding site (RBS), and a stem domain which is essential for viral/cell membrane fusion. The currently licensed influenza vaccines (inactivated virus- or recombinant HA-based) focus on eliciting neutralizing Abs that target the HA head domain, interfere with receptor binding, and block the entry of virus into host cells [8,9,10]. The globular head of the HA is also the most immunodominant part of the virus and therefore is subject to ongoing positive selection as the virus evolves to evade the host immune system [11,12]. A relatively small number of amino acid mutations in the IAV head are sufficient to produce a new strain that is resistant to immunity against its predecessor, indicating that most antibodies against IAV are highly strain specific. The more conserved HA stem region exhibits low intrinsic immunogenicity, but infection or currently licensed seasonal influenza vaccines can still boost pre-existing memory responses to the HA stem in humans [13]. Broadly neutralizing antibodies (bnAbs) that target the HA stem and bind to multiple strains, or even multiple subtypes, of IAVs have been identified in human memory B cells [14,15]. Studies focused on characterization of HA stem-specific bnAb response to currently available seasonal vaccines have demonstrated cross-neutralizing Abs titers against H5N1 [16,17,18], as well as cross-group HA-specific B cells [19]. Moreover, adult humans exposed to novel influenza strains (e.g., 2009 pandemic A(H1N1)pdm09 or avian influenza vaccination/infection) generated a relatively abundant HA stem-specific response due to expansion of memory B cells targeting conserved regions shared by seasonal strains [20,21,22,23,24]. As a result of multiple exposures to IAV, the adult human MBC compartment typically has a broad repertoire of anti-influenza specificities and is continuously shaped by seasonal influenza virus strains [25].

The vast pool of potential BCR variable region sequences means that different individuals rarely share antibodies with similar sequences. However, recent MBC repertoire studies have shown that common pathways of antibody development and maturation can be engaged in different individuals, leading to “public” Ab classes with molecular characteristics and binding epitopes shared by several donors, including a number of public bnAb that target relatively conserved HA epitopes [26,27].

Understanding cross-reactive immunity, including MBC and bnAb evolution, is important for assessing the state of population immunity against emerging influenza viruses, improving influenza vaccines, and the development of therapeutic antibodies. However, there is limited information on HA-specific memory B cell repertoire in vaccinated people. The low frequency of HA-specific B cells (usually <0.05% of lymphocytes) makes these studies challenging. However, various recently developed single-cell RNA sequencing (scRNA-seq) platforms, in conjunction with enrichment/sorting methods providing Ab paired heavy and light chains, have made the study of antigen-specific BCR repertoire faster and more feasible [28,29,30].

In this study, we used high-throughput single-cell sequencing of HA probe-positive MBCs collected from an individual with repeated seasonal influenza vaccination but no recorded exposure to avian influenza virus antigen. We detected at least two cross-group bnAb clonotypes within the MBC BCR repertoire, with specificity to multiple HAs, including those from avian influenza strains.

## 2. Materials and Methods

### 2.1. Clinical Sample

The study was conducted in accordance with the Declaration of Helsinki and approved by the Institutional Review Board of Emory University (protocol code CR2_IRB00045947, date of approval 24 July 2012). Informed consent was obtained from the subject involved in the study. A peripheral blood mononuclear cell sample was collected from an adult (42 years of age, African American female) during the 2012–2013 influenza season, 14 days following commercial TIV vaccination. The donor was a healthcare professional and had been receiving an annual seasonal influenza vaccine since 1991 (self-report) and did not report any previous influenza virus infection or immunization with avian vaccines. The donor had asthma, high blood pressure, and seasonal allergies and was taking Amlodipine for high blood pressure and Singulair (montelukast sodium)/Symbicort (budesonide and formoterol) for asthma.

### 2.2. Recombinant HAs for B Cell Staining/Sorting and Biolayer Interferometry (BLI) Testing

The in-house-produced recombinant HAs (recHA) for B cell staining/sorting (Table S1) were codon optimized and expressed in the baculovirus–insect cell expression system as trimers with a C-terminal foldon and His-tag as previously described [31,32]. Secreted soluble trimeric recHA protein was recovered from the cell culture supernatant by tangential flow filtration (Pall Corporation/Cytiva, Marlborough, MA, USA) through either a 30 kDa membrane (Cytiva, cat # OS030T12) for the HA ectodomain or 10 kDa molecular weight cutoff membrane (Cyntia, cat # OS010T12) for HA stems, metal affinity chromatography utilizing Ni-NTA superflow beads (Qiagen, Germantown, MD, USA, cat # 30430) and a Ni-Sepharose His-Trap FF 5 mL column (GE Healthcare, Waukesha, WI, USA, cat # 17-5318-02). The final protein purification step utilized size exclusion chromatography via an AKTA Pure chromatography system (GE Healthcare) and Superdex 200 16/60 column (GE Healthcare). Sialic acid binding was prevented with a Tyr91Phe (Y91F) RBS mutation in the A(H1) and the equivalent Y98F mutation in the A(H3) recHA probes [23,33]. RecHAs for BLI testing were prepared either from a baculovirus expression system or from HEK293 cells (Table S1).

### 2.3. Fluorescence-Activated Cell Sorting of HA-Specific B Cells

B cells were enriched from PBMCs with EasySep Human B cell isolation kit (Stem Cell, Cambridge, MA, USA, cat # 17954) and stained. Anti-CD3-BV605 (BioLegend, San Diego, CA, USA, cat # 317322), anti-CD14-BV605 (BD, Franklin Lakes, NJ, USA, cat # 564054), anti-CD16-BV605 (BD, cat # 563173), anti-CD19-PE (BD, cat # 555413), anti-IgD-PerCP-Cy5.5 (BD, cat # 561315), and Zombie Violet Fixable Viability Kit (Biolegend, cat # 423114) were used for staining. Receptor-binding site mutated A(H1N1)pdm09, A/California 07/2009 [GISAID, EPI177294] Y91F and A(H3N2), A/Texas/50/2012 [GISAID, EPI2318462] Y98F recHAs were conjugated to DyLight 488 (Thermo Fisher, Carlsbad, CA, USA, cat # 46403) and DyLight 650 (Thermo Fisher, cat # 62266), respectively, and used for staining. HA-specific B cells (live CD3-CD14-CD16-CD19+A/California HA+ and A/Texas HA+, both single HA+ and double HA+ cells; 0.17% of CD19+ B cells) were sorted (count 11,000 cells) using a BD FACSAria™ II and subjected to Chromium Single Cell 5′V(D)J protocol (10x Genomics, Pleasanton, CA, USA). The resulting library was sequenced with Illumina (San Diego, CA, USA). Number of productive paired sequences recovered: 3038.

### 2.4. Bioinformatic Analysis of BCR Repertoire Data

Dandelion (v0.3.3) [34] was used to process and analyze the scVDJ-seq data. Each sample was pre-processed and filtered to retain only “high confidence” contigs using the containerized workflow, which involves (1) VDJ gene reannotation using IgBLASTn (v1.21.0) [35]; (2) V gene allele reassignment with TIgGER (v1.1.0) [36]; (3) constant region reassignment; and (4) mutation quantification using SHazaM (v1.2.0) [37]. The resulting objects were further processed by removing contigs lacking values for the V-gene calls, J-gene calls, or amino acid junction annotations. Finally, the objects were filtered to retain only cells with a single productive heavy-chain/light-chain pair.

Sequences were grouped into clonotypes using the Change-O (v1.3.0) [37] clonotype calling pipeline implemented through Dandelion via a Singularity container. This workflow uses SHazaM and Change-O to infer a dataset-specific distance threshold and partition sequences based on shared IGHV genes, IGHJ genes, and junction region lengths prior to hierarchical clustering into clonal groups. For phylogenetic analysis, we retained heavy-chain records with a clonotype assignment, a constant-region assignment, and a single unambiguous constant-region call. Clone-specific germline-aligned nucleotide sequences were reconstructed with createGermlines in Dowser (v2.2.0) [38]. The human IMGT V, D, and J reference snapshot (downloaded on 1 October 2024) [39] was used as the base reference. Its IGH V-reference collection was updated with the V-allele sequences in the Day-14 TIgGER genotype FASTA; the IMGT D- and J-references were retained. The TigGER genotype-assigned V-gene field, v_call_genotyped, was supplied explicitly during germline reconstruction. The resulting D-masked germline alignment was used during clone formatting. Clones were formatted from heavy-chain nucleotide alignments using the sequence identifier and Change-O clone identifier, with constant-region assignment retained as a trait, duplicate count retained as a numeric field, and a minimum clone size of three sequences. Identical observed nucleotide sequences were collapsed during clone formatting. Maximum-likelihood lineage trees and internal ancestral nodes were inferred using IgPhyML (v2.0.0) [40] through Dowser. IgPhyML was run with a single heavy-chain partition, FCH hotspot and coldspot motifs, and an ancestral-sequence reconstruction cutoff of 0.95. Random-number seeds were fixed at 15,244 for clonotype 152_44 and 11,701 for clonotype 117_1. Redundant internal nodes were collapsed after inference, and branch lengths were scaled to represent numbers of nucleotide mutations. Ambiguous ancestral nucleotide states were retained as IUPAC ambiguity codes rather than resolved to a single base. Protein sequence alignments were performed with Geneious Prime (v2025.0.2, Boston, MA, USA).

Previously reported conserved motifs among influenza A-specific antibodies were compiled from the literature [41,42,43,44,45] and queried against the dataset of the paired heavy- and light-chain sequences identified from the HA-specific B cells. Clonotypic clustering of the 117_1 sequences was performed with 5561 anti-influenza antibodies collated by Wang et al. [46]. A total of 5524 heavy-chain BCR sequences with annotated heavy-chain V and J gene calls were filtered from the Wang et al. dataset and analyzed together with the 117_1 antibody clonotype, comprising approximately 99 clonal members. Sequences were assigned to the same clonotype if they shared the same IGHV and IGHJ gene annotations, had identical CDR3 lengths, and exhibited ≥80% amino acid sequence identity within the CDR3. The clonotyping workflow is available on GitHub at https://github.com/CDCgov/MVVT/ (accessed on 12 August 2026).

### 2.5. Expression and Primary Testing mAbs

We used a linear cassette-based monoclonal antibody (mAb) cloning/expression method [47]. Briefly, the paired heavy (VH) and light (VL) variable chain sequences for mAbs were obtained from Twist Biosciences (South San Francisco, CA, USA). During overlapping PCR, the VH and VL sequences were joined to the corresponding heavy and light constant regions into a linear expression construct containing promoter, self-cleaving peptide, and poly(A) signal for expression of IgG1 mAb with kappa or lambda light chains. PCR was done using the Platinum™ SuperFi II DNA Polymerase (Thermo Fisher, cat # 12361010). The expression construct was purified with QIAquick PCR and Gel Cleanup Kit (Qiagen, Englewood, NJ, USA, cat # 28506) and used for transfection of Expi293F cells (Thermo Fisher, cat # A14635). The supernatants were harvested 5 days post-transfection, and the concentration of mAbs was determined using biolayer interferometry (BLI). The mAb-containing supernatants were further tested for binding to A(H1), A(H3) HA ectodomain trimers as well as Group 1 and Group 2 HA stem constructs by BLI.

### 2.6. Unmutated Common Ancestor (UCA) and Branch Point (BP) mAb Design

Heavy chains 152_44 UCA_H and 117_1 UCA_H were designed as model-informed, germline-reverted variable region sequences guided by the computational germline and ancestral reconstructions described above (Section 2.4). For construction of 152_44 UCA_H_1, CDRH3 was used from the clonotypes’ representative mAbs n2. For construction of 117_1 UCA_H_1, we used CDRH3 of the clonotypes’ representative mAb n10, the most frequently utilized in the clonotype. To construct the CDRH3 of 117_1 UCA_H_2 and 117_1 UCA_H_3, single substitutions H108S and H108T, respectively, were introduced into the CDRH3 of mAb n10. For construction of 117_1 UCA_H_4, CDRH3 of another clonotypes’ representative mAb n27 was utilized. The UCA and BP mAbs had similar light chains within the 152_44 and 117_1 mAb groups, which were constructed as germline sequences with CDR3 from the corresponding node 1 mAbs. The N55T mutations were introduced into 152_44 UCA_H_1.

### 2.7. Kinetics and KD Determination

mAb quantitation and binding kinetics were performed by biolayer interferometry (BLI) using Octet 8 (Sartorius, New York, NY, USA). The quantitation assay was performed with Protein A biosensors (Sartorius, cat # 18-5012). The standard curve was generated using ProA Calibrator (Sartorius, cat # 18-1118). Kinetic analyses of mAb binding to recombinant HAs (except H5) were performed at 25 °C as follows. Full length trimeric HA (expressed with His tag) was captured on HIS1K biosensors (Sartorius, cat # 18-5121) at 5 or 7.5 µg/mL (28 or 42 nM) depending on the HA in a kinetics buffer (KB, Sartorius, cat # 18-1105) for 90 s. The loaded biosensors were washed for 60 s in sample diluent (SD, Sartorius, 18-1104) and exposed to seven concentrations of mAb for 300 s. Two-fold serial dilutions of mAb were prepared in SD and loaded at concentrations from 1.25 µg/mL to 80 µg/mL (8 to 533 nM). For mAb-H5 HA binding, the following protocol was used. mAb was captured on Protein A biosensors (Sartorius, 18-5012) at 7.5 µg/mL (50 nM) for 90 min. The loaded biosensors were washed in KB for 60 s and exposed to seven concentrations of HA for 300 s. Two-fold serial dilutions of HA were prepared in KB and loaded at concentrations from 0.8 µg/mL to 50 µg/mL (4.3 to 278 nM). The K_D_ values were calculated by Steady State analysis with Octet Analysis Studio 12.2.

### 2.8. High-Content Imaging Neutralization Test (HINT) for Seasonal Influenza Strains

The assay was applied to test monoclonal antibodies against influenza viruses [48]. It is based on a single infection cycle which is achieved by omitting trypsin from the virus growth media. Briefly, viruses were pre-titrated on MDCK-SIAT1 cells (Sigma, St. Louis, MO, USA, cat # CB_05071502) in 96-well black clear bottom plates (Corning, Glendale, AZ, USA, cat # 3603). Plates were incubated for 18–20 h. Cell monolayer was fixed with ice-cold ethanol and acetic acid (95:5). Virus-infected cells were detected by immunostaining with mouse anti-IAV nucleoprotein (NP) monoclonal antibodies (1:1000; IRR, FR-794) followed by anti-mouse IgG antibody conjugated to Alexa Fluor^TM^ 555 (1:1000; Thermo Fisher, cat # A221424). Plates were scanned with a Celigo imager (Revvity, Waltham, MA, USA). Generated data was used to determine the dilution factor to achieve 2 × 10^4^ infectious units per milliliter. For virus neutralization, serially two-fold diluted monoclonal antibodies (50 µL) in range 0.02–10 µg/mL were preincubated with diluted influenza virus (50 µL) for 1 h at room temperature. Incubation of virus–antibody mix was performed in the wells of a 96-well black clear bottom plate. Cell suspension 3 × 10^6^/mL was added (50 µL) to each well. Plates were incubated for 18–20 h and immunostained for influenza virus antigen, as described above. The results, after plate scanning (Celigo imager), were used to calculate EC_50_ by utilizing in-house JASPR (v5.0) software.

### 2.9. Microneutralization Assay for A(H5N1)

The assay was conducted at BSL-3E and performed as previously described [49,50] using egg-propagated A(H5N1) A/Texas/37/2024 (GISAID, EPI3171488) isolated from a human infection during the A(H5N1) outbreak in dairy cows in 2024. Antibodies (at starting concentration 1 mg/mL) were serially diluted 2-fold and incubated at 37 °C for 1 h with one hundred 50% tissue culture infectious dose (TCID50) of influenza viruses per well. The virus–antibody mixture was then used to infect 1.5 × 10^4^/well MDCK cells and incubated overnight. The plates were fixed with cold 80% acetone and washed, and the presence of viral nucleoprotein quantified by enzyme-linked immunosorbent assay (ELISA) using mouse anti-IAV NP mAb (in-house, CDC produced) followed by anti-mouse IgG (HRP-conjugated) (SeraCare, Milford, MA, USA, cat # 5220-0339) [51]. Plates were scanned with SpectraMax ABS Plus (Molecular Devices, SanJose, CA, USA) and microneutralization titers were defined as the reciprocal of the highest antibody dilution that showed 50% neutralization.

### 2.10. Viruses

Wild-type influenza strains were obtained from the Centers for Disease Control and Prevention. The viruses were propagated (see passage history in Table S2), and virus titers were determined as the mean TCID50 per milliliter.

## 3. Results

To assess cross-reactive humoral immune response to immunization, we analyzed the BCR repertoire of influenza HA-specific MBCs from peripheral blood mononuclear cells (PBMCs) of a female healthcare professional during the 2012–2013 influenza season. PBMCs collected at day-14 post-vaccination were enriched for B cells by negative selection, stained with A(H1) and A(H3) HA probes, and sorted for HA-positive (including single positive and double positive) CD19 cells (Figure S1 and Section 2.3). From sorted HA-specific MBCs, we sequenced paired heavy and light chains using the single-cell 10x Genomics Chromium platform with Illumina sequencing [30]. We performed bioinformatic analysis, including V(D)J gene usage, clonotyping, clonotype ranking and phylogenetic tree construction for lineage inference.

The representative mAbs from the twenty most expanded class-switched lineages were expressed [47] and screened for binding to A(H1) and A(H3) HA ectodomain trimers as well as Group 1 and Group 2 HA stem constructs (Table S1) by biolayer interferometry (BLI). We identified three lineages that were binding to A(H1) and A(H3) HAs and Group 1 and Group 2 HA stems: 152_44, 117_1 and 256_4 (Table S3). 117_1 and 152_44 were among the most expanded class-switched lineages in the repertoire. The molecular signatures of 117_1 and 256_4 demonstrated high similarity (same germline genes, with highly similar CDR3 for heavy and light chains, but one amino acid difference in the length of CDRH3) and were considered as one signature. The clonotypes were grouped for HA binding and virus neutralization experiments (117_1/256_4 cluster) (Table 1 and Table 2). Representative mAbs from cross-reactive 152_44 clonotype and 117_1/256_4 cluster (Figure S2) were compared with previously described HA-specific bnAbs for binding to Groups 1 and 2 HA (Table 1) and virus neutralization (Table 2a,b). The molecular signatures of the 152_44 and 117_1 clonotypes were searched against the previously reported conserved motifs within IAV-specific Abs compiled from the literature [41,42,43,44,45].

**Figure 1:**
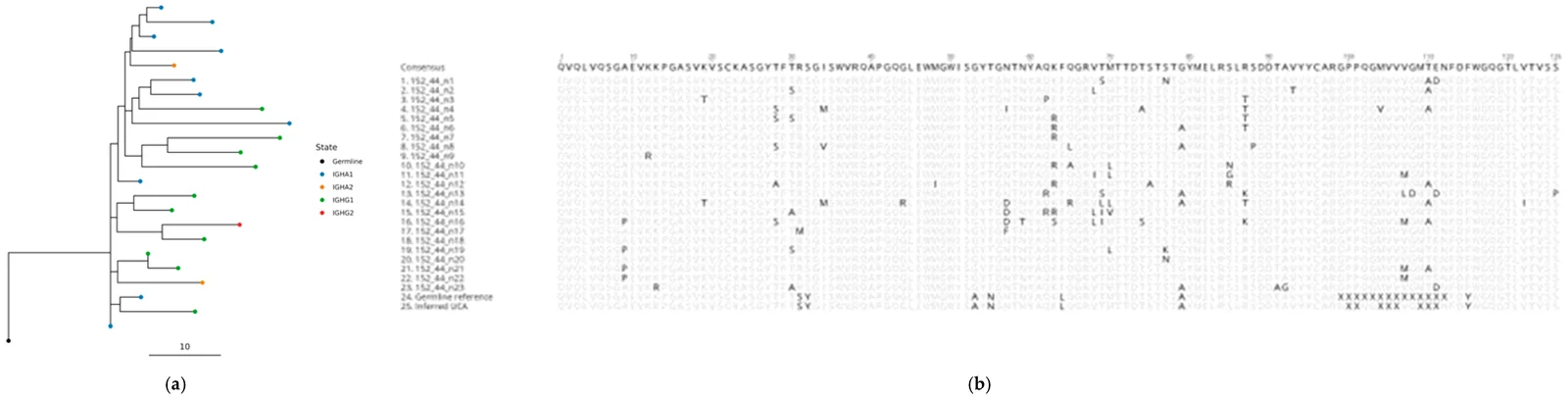

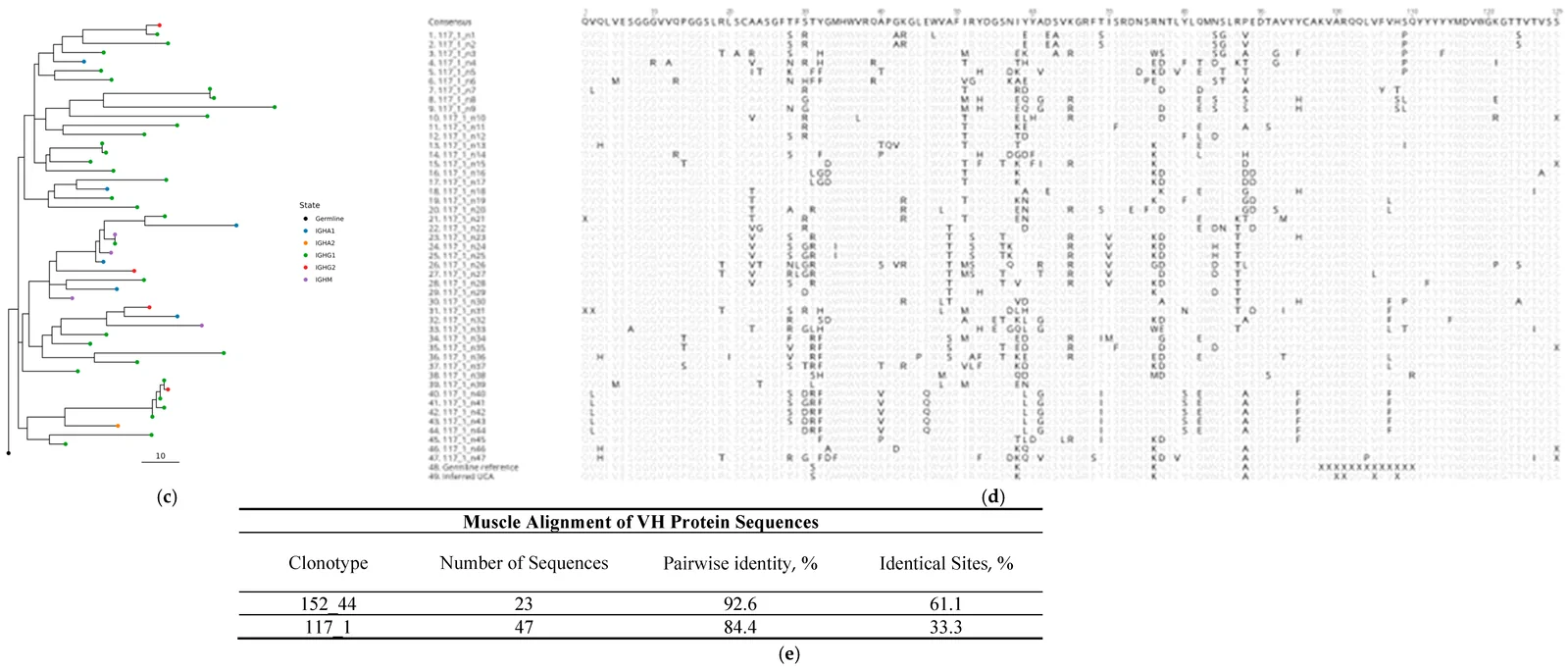
VH alignment and phylogenetic trees of 152_4 and 117_1 clonotypes. The phylogenetic trees of VH nucleotide sequences for 152_44 (**a**) and 117_1 (**c**) clonotypes were generated as described in Section 2.4. State: Ab isotype. Scale bar indicates number of nucleotide mutations along branches. VH protein sequence alignments for members of 152_44 (**b**) and 117_1 clonotypes (**d**) (named as clonotype name followed by node (n) number) were done with Geneious (2026.1) Prime (Boston, MA, USA). Residues differing from the consensus sequence are highlighted in black. Percents of pairwise identity and identical sites for the VH protein sequences for the clonotypes were calculated with Geneious Prime (v2025.0.2) (**e**).

The clonotype 152_44 (VH1-18/DH3-22/JH4 & VK3-20/JK2) [Figure 1a,b] possesses the VH1-18 QXXV motif found in a HA-stem-binding cross-group reactive antibody family [42]. All tested members of 152_44 demonstrated binding to both A(H1) HA and A(H3) HA from historical and contemporary strains (Table 1) but neutralized primarily A(H3N2) (2009–2017) viruses. All tested members of 152_44 demonstrated binding to both A(H1) HA and A(H3) HA from historical and contemporary strains (Table 1) but neutralized primarily A(H3N2) (2009–2017) viruses. In fact, only two of the seven tested members of this family, namely 152_44_n11 and n23, were able to weakly neutralize one or two A(H1N1)pdm09 isolates, respectively (Table 2a). The A(H1)/A(H3) HA binding and A(H3N2) neutralization activities were comparable with the properties of the highly potent Fi6v3 [19], MEDI8852 [52], and CR9114 [53] mAbs. Additionally, the 152_44 mAbs demonstrated affinity to A(H5), A(H7), A(H9) and A(H13) HAs at nanomolar levels, comparable to binding affinities of Fi6v3 and MEDI8852 (Table 1). Moreover, one of the four tested 152_44 mAbs (152_44_n11, one of the two antibodies that demonstrated neutralization of A(H1N1)pdm09) neutralized bovine A(H5N1) with a microneutralization (MN) titer comparable to that of Fi6v3 (Table 2b).

We modeled the evolution of the 152_44 lineage by constructing a phylogenetic tree for the heavy chain of the 152_44 lineage at the nucleotide level (Figure 1a). We expressed the unmutated common ancestor (152_44 UCA) mAb and compared it to the lineage’s representative mAb 154_44_n11, and to the N55T single mutation intermediate (152_44 UCA_N55T), for binding to HAs from various seasonal A(H1) and A(H3) strains by BLI (Figure S3). Overall, 152_44 UCA mAb demonstrated very weak binding (especially with A(H1) HAs) compared to 152_44_n11, except for A/Darwin/9/21(H3N2). The introduction of the N55T [54] single mutation into 152_44 UCA significantly improved binding to the HAs (Figure S3).

The second clonotype 117_1 (VH3-30/DH6-13/JH6 and VK2-28/JK5) [Figure 1c,d], which exhibited a signature of the 117_1/256_4 cluster, did not match the previously described functional motif or sequence signatures of broadly cross-reactive anti-influenza antibodies. Although many bnAbs utilizing the IGHV3-30 germline gene have been identified, including Fi6v3 [19], 3I14 [14], PN-SIA28 [55,56], none of the antibodies collated by Wang et al. [46], including those that use the IGHV3-30 gene, meet criteria for clonotypic clustering with 117_1, as they have different CDR3 lengths and sequences and do not share IGHJ and/or light chain V and J usage (Table S4).

The seven representative mAbs from the 117_1/256_4 cluster demonstrated affinity to A(H1) and A(H3) viruses from historical and contemporary strains (Table 1) and neutralized all tested A(H1N1)pdm09 (2009–2023) and A(H3N2) (2009–2017) viruses (Table 2a). The binding and neutralization activities were comparable with the properties of the highly potent Fi6v3, MEDI8852 [52] and CR9114 [53] mAbs (Table 1 and Table 2a). 117_1/256_4 mAbs demonstrated affinity to A(H5), A(H7), A(H9) (at nM level) and again were comparable to Fi6v3 and MEDI8852 (Table 1). Moreover, two of the four tested mAbs neutralized bovine A(H5N1), as did Fi6v3 (Table 2b). We modeled the evolution of the 117_1 lineage’s heavy chain in the phylogenetic tree (Figure 1c) and expressed the unmutated common ancestor (UCA) and nine branch point (BP) mAbs. The 117_1 UCAs demonstrated substantial binding to A(H1) and A(H3) HAs (A/Brisbane/02/18 and A/Singapore/INFIMH-16-0019/2016, respectively), and BP Abs demonstrated binding comparable to the 117_1 node Abs (Figure S4).

## 4. Discussion

We performed IAV HA-specific MBC immunoprofiling for an adult who had been annually immunized with seasonal influenza vaccines for at least 10 years prior to sample collection during the 2012–2013 influenza season. At the time of sample collection, the person had controlled underlying health conditions and no record of recent influenza infection or participation in avian vaccine trials. However, undocumented or asymptomatic seasonal influenza exposure could not be ruled out. In the blood sample, we identified three anti-influenza memory B cell lineages with broad cross-reactivity against multiple strains of A(H1N1) and A(H3N2) subtypes as well as to avian subtypes that have caused human infections (A(H5), A(H7), and A(H9)). The HA binding affinities of representative mAbs from the naturally occurring lineages 117_1, 256_4 and 152_44 were comparable to the affinities of the highly potent gold-standard anti-HA bnAb Fi6v3, MEDI8852 and CR9114 that have been artificially generated and optimized for breadth and affinity [19,52,53].

The 152_44 clonotype displayed the molecular signature of a VH1-18 QXXV multi-donor cross-group binding immunoglobulin class that was initially identified in a subset of vaccinees from an A(H5N1) vaccine trial [42]. Andrews et al. demonstrated that the H7N9 vaccine induced a more significant level of VH1-18 QXXV responses (compared to A(H5N1) vaccine) with preferential binding to Group 2 strains [26]. Additionally, the class heavy-chain sequence signature ^53^Y^54^T^98^QXXV was also found in post-TIV (trivalent inactivated influenza vaccine) and healthy normal donor datasets [42], and Wu Y et al. isolated CT149 (VH1-18 QXXV/VK3-20) mAbs from a convalescent patient infected with pandemic A(H1N1)pdm09 in 2009. CT149 neutralized all tested Group 2 and some Group 1 IAV by inhibiting low pH-induced, HA-mediated membrane fusion [57].

In our study all expressed 152_44 mAbs bound to multiple A(H1) HAs. However, only two of the seven tested members of this family, specifically 152_44_n11 and n23, were able to weakly neutralize one or two A(H1N1)pdm09 isolates, respectively (Table 2a). All the expressed 152_44 mAbs efficiently neutralized multiple A(H3N2) viruses. This agrees with the data from Andrews SF et al. demonstrating that VH1-18 QXXV mAbs preferentially bound to group 2 HAs, and while binding A(H1N1)pdm09 HA, the mAbs did not provide in vitro neutralization of the same strains. Nevertheless, the VH1-18QXXV Abs enhanced the survival of mice challenged with A(H1N1)pdm09 and even A(H7N9) strains [26].

Broad-spectrum influenza protection depends on the presence of bnAb precursors within the B cell repertoire and the potential of the vaccine or virus to engage the precursors. We identified the public VH1-18 QXXV clonotype in an extensively vaccinated individual and, additionally, we found in the same vaccinee a novel bn clonotype 117_1, expanding the known repertoire of highly potent and cross-reactive antibodies. Moreover, the third cross-reactive clonotype found in the vaccinee (256_4) demonstrated a molecular signature highly similar to that of 117_1.

Although adults are repeatedly exposed to and generate immune responses to many strains of influenza during their life, the nature of an individual’s immune response is strongly influenced by the influenza strain to which they are first exposed, a process known as “imprinting”. Subsequent infections or vaccinations lead to preferential amplification of responses to more conserved epitopes that are shared between the original and novel strains. In the case of the healthcare worker in this study, three of the 20 most expanded class-switched B cell lineages were broadly cross-reactive, perhaps as a result of multiple vaccinations received over previous decades. However, further studies will be required to understand the normal background of broadly cross-reactive antibodies in adult humans.

Modeling bnAb evolution is critical for understanding immune response against rapidly mutated IAV and advancing universal vaccine design. The UCA of the 152_44 lineage demonstrated very weak binding to A(H1) and A(H3) HAs. Incorporating the N55T substitution (found in all discovered sequences in this donor) significantly improved the binding. This result agrees with data from Ray et al. on the importance of N55T substitution for the binding of VH1-18 QXXV Abs to HA [54].

In contrast to 152_44, the 117_1 UCA demonstrated good binding to A(H1) HA and even better affinity to A(H3) HA. Members of this clonotype acquired higher levels of somatic mutations and sequence variability compared to 152_44, possibly the result of a longer period of evolution (Figure 1e). Modeling of B cell 117_1 and 152_44 clonotype natural evolution can help to better understand the elicitation of HA stem-targeted B cells to Group 1 and Group 2 influenza subtypes.

Conventional seasonal influenza vaccines typically induce antibody responses that target the immunodominant but highly mutable head domain, meaning that while they can provide effective immunity, they need to be regularly updated. Broadly neutralizing antibodies (bnAbs) target relatively conserved regions of HA and can cover different levels of IAV diversity. HA stem-targeting Abs can block infection by interfering with HA cleavage and the proper conformational change in HA trimer, required for membrane fusion, or affect infection via cell-mediated mechanisms such as ADCC [58,59].

The HA stem-conserved region is typically subdominant, and the response to the HA stem usually requires immunogen/vaccination platforms specifically targeting the domain. However, an increasing number of studies have identified bnAb in adults. While such bnAb are typically minor components of the anti-influenza antibody repertoire [60,61], in this donor we found that two of the three most expanded class-switched lineages in the MBC repertoire produced cross-group binding Abs even capable of reacting with non-seasonal IAV such as the A(H5N1) and A(H7N9) viruses. Although this donor had no history of exposure to non-seasonal IAV, as a healthcare professional she had received annual influenza vaccines for many years, potentially recalling broadly cross-protective anti-influenza MBC response. It is noteworthy that in spite of her presumably relatively high risk of exposure to infectious disease, the donor had no history of IAV disease at the time of sampling and had only been diagnosed with an IAV once to date (2024, mild symptoms, self-report), suggesting this broadly cross-reactive MBC repertoire may be associated with effective protection.

Pre-existing humoral immunity against bovine A(H5N1) influenza virus has been described in H5-naïve adults [62], and there are limited publications on cross-reactive immunity against avian A(H5N1) [16,17,18] and cross-group HA-specific immunity to seasonal vaccination [19].

## 5. Conclusions

Our work provides additional evidence that seasonal influenza vaccines may support the expansion of IAV cross-reactive B cells, potentially mitigating the risk of the emerging influenza strains.

## 6. Limitations of the Study

Our work has several limitations.

1. The study included only one participant, a 42-year-old female healthcare worker, whose sample was collected 14 days after vaccination. Information on exposure to influenza antigens was self-reported by the donor.
2. No pre-vaccination sample was tested, and we only can speculate that the cross-binding lineages were expanding to vaccination based on lineage size ranking.
3. Serological samples were not available for the donor.
4. Our conclusion that the 117_1 lineage has epitopes on the stem is based on affinity to the stem construct. We did not perform epitope mapping.
5. There are multiple limitations of inferred ancestors in B cell clonal lineages, especially modeling of the CDR3 region due to variable trimming, non-templated nucleotide addition at the V(D)J junction, and somatic hypermutation during lineage evolution. Additionally, we developed the model with a focus on heavy chain only. Finally, our study did not include comparisons with unvaccinated individuals, less frequently vaccinated adults, individuals with prior natural infection, or younger adults with fewer prior exposures; thus, the findings should be interpreted as evidence of association rather than causation.

## Figures and Tables

**Table 1:** HA binding (dissociation constant K_D_, nM) by mAbs from 152_4 clonotype and 117_1/256_4 cluster. Representative mAb from 152_44 and 117_1/256_4 were tested for binding to recombinant HAs (Table S1). Broadly neutralizing cross-group HA stem-binding mAb Fi6v3 [19], MEDI8852 [52] and CR9114 [53] were used for comparison. The testing was done by BLI (biolayer interferometry). Dissociation constant (K_D_ ± K_D_ Error) was calculated with steady-state analysis (Octet Analysis Studio 12.2) from seven dilutions. mAbs are color-coded according to the K_D_ (nM): less than 10 nM (purple), 10–60 nM (green) and more than 60 nM (blue). N: negative. Gray: not tested. See Table S1 for full names of the recombinant HAs used for the testing. All tested strains, except A/C/4/2009 (H1N1pdm) and A/HK/1/1968 (H3N2), could not have been encountered by the donor.

			152_44							117_1/256_4							Control		
	Subtype	Strain	n7	n11	n14	n20	n18	n23	C-134	n10	n16	n19	n32	n27	256_4-pr	256_4-C	Fi6v3	MEDI8852	CR9114
Group 1	H1N1	A/PR/8/1934	24 ± 2		110 ± 9.3	75 ± 4.8		49 ± 1.6				29 ± 2.2	9 ± 0.4			13 ± 0.8	3 ± 0.2	11 ± 0.9	
	H1N1pdm09	A/C/4/2009			14 ± 2.8	5.4 ± 0.7		6 ± 0.9				17 ± 2.3	5 ± 0.4			10 ± 0.95	6 ± 0.5	17 ± 1.5	
	H1N1pdm09	A/M/45/2015			24 ± 4	5 ± 0.4		4 ± 0.3				10 ± 0.3	3 ± 0.2			5 ± 0.2	3.8 ± 0.5	8.3 ± 0.4	
	H5N1	A/A/1/2005	7.4 ± 0.9	6.7 ± 0.4	9.2 ± 0.9	9.2 ± 0.6	8.2 ± 0.7	8.1 ± 0.6	7.2 ± 0.6	11 ± 0.8	66 ± 5.6	190 ± 35	130 ± 14	6 ± 0.5	86 ± 2	7.8 ± 1	58 ± 9	8.5 ± 0.8	11 ± 0.1
	H5N1	A/Q/1A/2005	3.6 ± 0.3	3.5 ± 0.3	5 ± 0.2	3.8 ± 0.3	3.3 ± 0.3	3.4 ± 0.4	3.6 ± 0.3	5.1 ± 0.5	21 ± 3	36 ± 4	40 ± 4.6	3.4 ± 0.3	15 ± 2.5	4 ± 0.5	13 ± 1.4	5 ± 0.3	4.4 ± 0.4
	H5N8	A/W/6/2014	16 ± 0.8	14 ± 0.4	42 ± 2.8	25 ± 1.2	21 ± 1.5	26 ± 1.7	19 ± 1.1	19 ± 0.9	91 ± 7.6	190 ± 9	41 ± 3.3	7.9 ± 0.3	72 ± 0.8	11 ± 0.8	50 ± 6.6	16 ± 1.2	13 ± 0.9
	H9N2	A/H K/1999			110 ± 16	12 ± 2		6.4 ± 0.7				81 ± 13	4.1 ± 0.7			4.2 ± 0.6	3.3 ± 0.3	4.5 ± 0.5	
	H13N9	A/D/68/2004	140 ± 11	75 ± 4	N	N		65 ± 7	58 ± 9			N	N	99 ± 6		88 ± 1.3	41 ± 3	100 ± 7	
Group 2	H3N2	A/HK/1/1968			6 ± 0.2	8 ± 0.3		3.4 ± 0.5				40 ± 1	16 ± 0.7			17 ± 0.7	2.9 ± 0.2	6.5 ± 0.4	
	H3N2	A/S/2016			32 ± 2	23 ± 1		17 ± 0.8				91 ± 1.4	21 ± 1			20 ± 1.2			
	H3N2	A/HK/45/2019			42 ± 3	32 ± 0.8		23 ± 1.4				110 ± 2	22 ± 0.5			28 ± 1	9.7 ± 0.8	19 ± 0.8	
	H3N2	A/D/9/2021			58 ± 3	39 ± 1		23 ± 0.8				120 ± 2	35 ± 1			40 ± 2	13 ± 0.9	28 ± 0.8	
	H7N9	A/NY/107/2003			12 ± 0.8	12 ± 0.4		10 ± 0.5				470 ± 40	100 ± 10			9.5 ± 0.6	4.8 ± 0.5	8.1 ± 0.4	

**Table 2:** Virus neutralization by mAbs from 152_4 clonotype and 117_1/256_4 cluster. (**a**) Representative mAb from 152_44 and 117_1/256_4 were tested for neutralization by HINT [48] with the indicated strains (Table S2). mAbs are color-coded according to the neutralization EC_50_ (µg/mL) average ± SD of three independent experiments: less than 0.2 (purple), 0.2–2 (orange), 2–5 (green) and 5–10 (blue). All tested strains (see Table S2 for full names), except A/C/7/2009 (H1N1pdm), A/P/16/2010(H3N2), A/V/361/2012 and A/T/50/2013(H3N2), could not have been encountered by the donor. (**b**) Representative mAb from 152_44 clonotype and 117_1/256 cluster were tested in duplicate within a single experiment for neutralization of bovine A/Texas/37/2024(H5N1). Geometric mean titers (GMT) were calculated. Previously published broadly neutralizing cross-group HA stem-binding mAb Fi6v3 [19] and CR9114 [53] were used for comparison.

(**a**)																	
		**152_44**							**117_1/256_4**							**Control**	
**Subtype/Type**	**Strain**	**n20**	**n11**	**C-134**	**n7**	**n14**	**n23**	**n18**	**n27**	**n16**	**n19**	**n32**	**n10**	**256_4-pr**	**256_4-C**	**Fi6v3**	**CR9114**
H1N1pdm09	A/C/7/2009	>10	>10	>10	>10	>10	>10	>10	0.48 ± 0.14	1.01 ± 0.3	2.46 ± 0.22	4.78 ± 2.42	0.53 ± 0.04	0.41 ± 0.07	0.28 ± 0.08	0.31 ± 0.08	0.08 ± 0.03
H1N1pdm09	A/B/2/2019	>10	5.37 ± 1.18	>10	>10	>10	3.75 ± 0.9	>10	1.43 ± 0.82	2.49 ± 1.14	2.16 ± 0.70	2.20 ± 1.55	1.31 ± 0.44	1.02 ± 0.33	0.99 ± 0.50	1.44 ± 0.52	0.30 ± 0.15
H1N1pdm09	A/W/588/2020	>10	>10	>10	>10	>10	>10	>10	4.90 ± 2.02	5.91 ± 3.46	>10	4.24 ± 1.20	5.02 ± 2.71	5.52 ± 1.73	3.94 ± 2.26	4.42 ± 2.20	1.02 ± 0.31
H1N1pdm09	A/H/70/2020	>10	>10	>10	>10	>10	>10	>10	2.06 ± 0.92	2.83 ± 1.52	3.64 ± 1.51	2.74 ± 1.67	1.93 ± 0.75	1.57 ± 0.66	1.16 ± 0.7	1.87 ± 1.32	0.37 ± 0.17
H1N1pdm09	A/R I/34/2024	>10	>10	>10	>10	>10	>10	>10	0.71 ± 0.23	0.86 ± 0.22	2.11 ± 0.11	1.27 ± 0.58	0.59 ± 0.05	0.52 ± 0.07	0.28 ± 0.06	0.47 ± 0.10	0.11 ± 0.02
H1N1pdm09	A/G/53/2024	>10	>10	>10	>10	>10	4.56 ± 0.42	>10	0.37 ± 0.07	0.56 ± 0.10	1.10 ± 0.12	0.59 ± 0.24	0.35 ± 0.02	0.29 ± 0.03	0.18 ± 0.03	0.24 ± 0.06	0.07 ± 0.02
H3N2	A/P/16/2010	0.29 ± 0.04	0.16 ± 0.05	0.15 ± 0.01	0.21 ± 0.02	0.10 ± 0.02	0.07 ± 0.01	0.16 ± 0.02	0.26 ± 0.01	0.28 ± 0.04	0.40 ± 0.05	0.35 ± 0.07	0.29 ± 0.03	0.69 ± 0.42	0.47 ± 0.3	0.17 ± 0.05	1.03 ± 0.24
H3N2	A/V/361/2012	1.89 ± 0.89	1.60 ± 1.01	1.06 ± 0.48	1.53 ± 0.64	1.18 ± 0.93	0.41 ± 0.14	1.22 ± 0.71	3.10 ± 1.71	1.56 ± 0.59	2.63 ± 1.53	2.68 ± 0.18	2.03 ± 0.72	4.7 ± 2.56	3.90 ± 1.58	1.43 ± 0.94	4.2 ± 1.08
H3N2	A/T/50/2013	2.13 ± 0.53	1.30 ± 0.47	1.32 ± 0.36	2.29 ± 0.68	1.28 ± 0.42	0.86 ± 0.24	1.57 ± 0.63	1.08 ± 0.39	0.86 ± 0.27	1.89 ± 0.49	0.89 ± 0.32	0.95 ± 0.32	2.09 ± 1.21	1.91 ± 1.53	1.33 ± 0.46	2.35 ± 0.65
H3N2	A/S/2014	4.24 ± 0.61	2.79 ± 0.51	2.62 ± 0.22	3.99 ± 0.48	2.91 ± 0.86	1.67 ± 0.06	2.79 ± 0.44	2.10 ± 0.49	1.73 ± 0.02	3.55 ± 0.76	2.35 ± 0.62	1.53 ± 0.17	3.56 ± 1.46	3.13 ± 1.72	1.81 ± 0.14	3.86 ± 0.24
H3N2	A/HK/2015	1.21 ± 0.06	0.9 ± 0.33	1.06 ± 0.11	1.55 ± 0.14	1.07 ± 0.14	0.53 ± 0.06	1.14 ± 0.14	0.66 ± 0.16	0.55 ± 0.10	1.37 ± 0.24	0.44 ± 0.06	0.54 ± 0.12	1.51 ± 0.90	0.85 ± 0.55	0.92 ± 0.27	1.33 ± 0.46
H3N2	A/W/33/2015	4.15 ± 0.33	3.08 ± 0.80	2.82 ± 0.52	3.92 ± 0.5	3.99 ± 0.28	2.24 ± 0.30	3.60 ± 0.45	2.20 ± 0.05	1.94 ± 0.43	4.43 ± 0.40	1.97 ± 0.38	2.25 ± 0.42	6.75 ± 1.95	2.14 ± 0.27	3.38 ± 0.46	6.39 ± 2.35
H3N2	A/P/46/2016	4.05 ± 0.99	2.80 ± 0.92	2.97 ± 0.66	3.13 ± 1.27	2.75 ± 0.89	2.12 ± 0.23	3.14 ± 0.34	1.65 ± 0.31	1.72 ± 0.38	3.85 ± 0.67	1.43 ± 0.17	1.43 ± 0.05	5.44 ± 1.73	1.88 ± 0.30	3.16 ± 0.71	4.32 ± 0.29
H3N2	A/S/2016	0.19 ± 0.04	0.25 ± 0.02	0.17 ± 0.02	0.23 ± 0.03	0.16 ± 0.01	0.13 ± 0.03	0.19 ± 0.03	0.21 ± 0.02	0.19 ± 0.02	0.72 ± 0.03	0.26 ± 0.03	0.23 ± 0.04	0.71 ± 0.02	0.22 ± 0.05	0.25 ± 0.04	0.68 ± 0.17
H3N2	A/L/50/2018	2.88 ± 0.85	4.29 ± 3.52	3.09 ± 1.65	2.80 ± 0.34	1.82 ± 0.19	2.77 ± 1.92	2.19 ± 0.22	3.38 ± 2.58	3.16 ± 2.43	3.58 ± 0.21	2.82 ± 2.12	3.32 ± 2.73	4.36 ± 0.55	3.27 ± 2.76	4.41 ± 3.39	4.32 ± 0.32
H3N2	A/L/49/2018	5.94 ± 1.40	3.56 ± 0.81	4.18 ± 0.94	5.02 ± 1.11	4.15 ± 0.64	2.98 ± 0.49	4.68 ± 1.14	3.23 ± 0.35	3.18 ± 0.53	3.56 ± 0.81	2.97 ± 0.45	3.24 ± 0.49	9.52 ± 0.67	3.23 ± 0.71	4.02 ± 0.61	6.02 ± 1.99
IBV	B/NC/2019	>10	>10	>10	>10	>10	>10	>10	>10	>10	>10	>10	>10	>10	>10	>10	>10
IBV	B/M/12/2019	>10	>10	>10	>10	>10	>10	>10	>10	>10	>10	>10	>10	>10	>10	>10	>10
(**b**)																	
**Clonotype/group**						**mAb**						**GMT**					
**152_44**						n7						40					
						n11						113					
						n20						28					
						n23						57					
**117_1/256_4**						n10						160					
						n16						57					
						n27						160					
						256_4-C						113					
**CONTROL**						Fi6v3						160					
						MEDI8852						453					

## Data Availability

Dataset available on request from the authors.

## Supplementary Materials

The following supporting information can be downloaded at https://www.mdpi.com/article/10.3390/vaccines14090742/s1, Figure S1: Flow cytometry gating for sorting HA-specific B cells; Figure S2: Protein sequence alignment of the cross-reactive mAbs used for BLI (K_D_) and MN testing; Figure S3: Binding of 152_44 UCA and UCA_N55T mAbs to A(H1) and A(H3) recHAs; Figure S4: Binding of 117_1 UCA and BP mAbs to A(H1) and A(H3) recHAs; Table S1: Recombinant HAs used in the study; Table S2: Seasonal influenza viruses used in the study; Table S3: HA-specific BCR repertoire of memory B cells: list of HA-specific clonotypes; Table S4: Comparison of the 117_1 clonotype to previously published bn antibodies with the IGHV3-30 gene (separately as excel file).

## Abbreviations

The following abbreviations are used in the manuscript:

MBC	memory B cell
HA	hemagglutinin
mAbs	monoclonal antibodies
NA	neuraminidase
IAVs	influenza A viruses
BCRs	B cell receptors
scRNA-seq	single-cell RNA sequencing
BLI	biolayer interferometry

## References

[1] Kumar B., Asha K., Khanna M., Ronsard L., Meseko C.A., Sanicas M. (2018). The emerging influenza virus threat: Status and new prospects for its therapy and control. Arch. Virol..

[2] Bouvier N.M., Palese P. (2008). The biology of influenza viruses. Vaccine.

[3] Gamblin S.J., Skehel J.J. (2010). Influenza hemagglutinin and neuraminidase membrane glycoproteins. J. Biol. Chem..

[4] Bi Y., Yang J., Wang L., Ran L., Gao G.F. (2024). Ecology and evolution of avian influenza viruses. Curr. Biol..

[5] Zheng B., Yang Y., Chen L., Wu M., Zhou S. (2022). B-cell receptor repertoire sequencing: Deeper digging into the mechanisms and clinical aspects of immune-mediated diseases. iScience.

[6] Inoue T., Kurosaki T. (2024). Memory B cells. Nat. Rev. Immunol..

[7] Wu N.C., Wilson I.A. (2020). Influenza Hemagglutinin Structures and Antibody Recognition. Cold Spring Harb. Perspect. Med..

[8] Trombetta C.M., Kistner O., Montomoli E., Viviani S., Marchi S. (2022). Influenza Viruses and Vaccines: The Role of Vaccine Effectiveness Studies for Evaluation of the Benefits of Influenza Vaccines. Vaccines.

[9] Coudeville L., Bailleux F., Riche B., Megas F., Andre P., Ecochard R. (2010). Relationship between haemagglutination-inhibiting antibody titres and clinical protection against influenza: Development and application of a bayesian random-effects model. BMC Med. Res. Methodol..

[10] Hobson D., Curry R.L., Beare A.S., Ward-Gardner A. (1972). The role of serum haemagglutination-inhibiting antibody in protection against challenge infection with influenza A2 and B viruses. J. Hyg..

[11] Kirkpatrick E., Qiu X., Wilson P.C., Bahl J., Krammer F. (2018). The influenza virus hemagglutinin head evolves faster than the stalk domain. Sci. Rep..

[12] Crowe J.E. (2019). Influenza Virus-Specific Human Antibody Repertoire Studies. J. Immunol..

[13] Tan H.X., Jegaskanda S., Juno J.A., Esterbauer R., Wong J., Kelly H.G., Liu Y., Tilmanis D., Hurt A.C., Yewdell J.W. (2019). Subdominance and poor intrinsic immunogenicity limit humoral immunity targeting influenza HA stem. J. Clin. Investig..

[14] Fu Y., Zhang Z., Sheehan J., Avnir Y., Ridenour C., Sachnik T., Sun J., Hossain M.J., Chen L.M., Zhu Q. (2016). A broadly neutralizing anti-influenza antibody reveals ongoing capacity of haemagglutinin-specific memory B cells to evolve. Nat. Commun..

[15] McCarthy K.R., Watanabe A., Kuraoka M., Do K.T., McGee C.E., Sempowski G.D., Kepler T.B., Schmidt A.G., Kelsoe G., Harrison S.C. (2018). Memory B Cells that Cross-React with Group 1 and Group 2 Influenza A Viruses Are Abundant in Adult Human Repertoires. Immunity.

[16] Gioia C., Castilletti C., Tempestilli M., Piacentini P., Bordi L., Chiappini R., Agrati C., Squarcione S., Ippolito G., Puro V. (2008). Cross-subtype immunity against avian influenza in persons recently vaccinated for influenza. Emerg. Infect. Dis..

[17] Sanz-Munoz I., Sanchez-Martinez J., Rodriguez-Crespo C., Concha-Santos C.S., Hernandez M., Rojo-Rello S., Dominguez-Gil M., Mostafa A., Martinez-Sobrido L., Eiros J.M. (2025). Are we serologically prepared against an avian influenza pandemic and could seasonal flu vaccines help us?. mBio.

[18] Zhang L., Behrens G.M.N., Kempf A., Nehlmeier I., Gartner S., Moldenhauer A.S., Graichen L., Happle C., Winkler M., Dopfer-Jablonka A. (2025). Neutralizing activity against bovine H5N1 HPAIV (clade 2.3.4.4b) in human plasma after seasonal influenza vaccination. Emerg. Microbes Infect..

[19] Corti D., Voss J., Gamblin S.J., Codoni G., Macagno A., Jarrossay D., Vachieri S.G., Pinna D., Minola A., Vanzetta F. (2011). A neutralizing antibody selected from plasma cells that binds to group 1 and group 2 influenza A hemagglutinins. Science.

[20] Wrammert J., Koutsonanos D., Li G.M., Edupuganti S., Sui J., Morrissey M., McCausland M., Skountzou I., Hornig M., Lipkin W.I. (2011). Broadly cross-reactive antibodies dominate the human B cell response against 2009 pandemic H1N1 influenza virus infection. J. Exp. Med..

[21] Thomson C.A., Wang Y., Jackson L.M., Olson M., Wang W., Liavonchanka A., Keleta L., Silva V., Diederich S., Jones R.B. (2012). Pandemic H1N1 Influenza Infection and Vaccination in Humans Induces Cross-Protective Antibodies that Target the Hemagglutinin Stem. Front. Immunol..

[22] Li G.M., Chiu C., Wrammert J., McCausland M., Andrews S.F., Zheng N.Y., Lee J.H., Huang M., Qu X., Edupuganti S. (2012). Pandemic H1N1 influenza vaccine induces a recall response in humans that favors broadly cross-reactive memory B cells. Proc. Natl. Acad. Sci. USA.

[23] Whittle J.R., Wheatley A.K., Wu L., Lingwood D., Kanekiyo M., Ma S.S., Narpala S.R., Yassine H.M., Frank G.M., Yewdell J.W. (2014). Flow cytometry reveals that H5N1 vaccination elicits cross-reactive stem-directed antibodies from multiple Ig heavy-chain lineages. J. Virol..

[24] Wheatley A.K., Whittle J.R., Lingwood D., Kanekiyo M., Yassine H.M., Ma S.S., Narpala S.R., Prabhakaran M.S., Matus-Nicodemos R.A., Bailer R.T. (2015). H5N1 Vaccine-Elicited Memory B Cells Are Genetically Constrained by the IGHV Locus in the Recognition of a Neutralizing Epitope in the Hemagglutinin Stem. J. Immunol..

[25] Lam J.H., Baumgarth N. (2019). The Multifaceted B Cell Response to Influenza Virus. J. Immunol..

[26] Andrews S.F., Joyce M.G., Chambers M.J., Gillespie R.A., Kanekiyo M., Leung K., Yang E.S., Tsybovsky Y., Wheatley A.K., Crank M.C. (2017). Preferential induction of cross-group influenza A hemagglutinin stem-specific memory B cells after H7N9 immunization in humans. Sci. Immunol..

[27] Finney J., Moseman A.P., Kong S., Watanabe A., Song S., Walsh R.M., Kuraoka M., Kotaki R., Moseman E.A., McCarthy K.R. (2024). Protective human antibodies against a conserved epitope in pre- and postfusion influenza hemagglutinin. Proc. Natl. Acad. Sci. USA.

[28] Smith M.J., Packard T.A., O’Neill S.K., Hinman R.M., Rihanek M., Gottlieb P.A., Cambier J.C. (2017). Detection and Enrichment of Rare Antigen-specific B Cells for Analysis of Phenotype and Function. J. Vis. Exp..

[29] Zheng G.X., Terry J.M., Belgrader P., Ryvkin P., Bent Z.W., Wilson R., Ziraldo S.B., Wheeler T.D., McDermott G.P., Zhu J. (2017). Massively parallel digital transcriptional profiling of single cells. Nat. Commun..

[30] Gao C., Zhang M., Chen L. (2020). The Comparison of Two Single-cell Sequencing Platforms: BD Rhapsody and 10x Genomics Chromium. Curr. Genom..

[31] Yang H., Carney P., Stevens J. (2010). Structure and Receptor binding properties of a pandemic H1N1 virus hemagglutinin. PLoS Curr..

[32] Mishina M., Cao W., Ende Z., Sharma S.S., Ray S.D., Kumari R., Kumar A., Shanmugasundaram U., Bohannon C.D., Ranjan P. (2025). Recombinant quadrivalent influenza vaccine (RIV) induces robust cell-mediated and HA-specific B cell humoral immune responses among healthcare personnel. Vaccine.

[33] Koutsakos M., Wheatley A.K., Loh L., Clemens E.B., Sant S., Nussing S., Fox A., Chung A.W., Laurie K.L., Hurt A.C. (2018). Circulating T(FH) cells, serological memory, and tissue compartmentalization shape human influenza-specific B cell immunity. Sci. Transl. Med..

[34] Suo C., Polanski K., Dann E., Lindeboom R.G.H., Vilarrasa-Blasi R., Vento-Tormo R., Haniffa M., Meyer K.B., Dratva L.M., Tuong Z.K. (2024). Dandelion uses the single-cell adaptive immune receptor repertoire to explore lymphocyte developmental origins. Nat. Biotechnol..

[35] Ye J., Ma N., Madden T.L., Ostell J.M. (2013). IgBLAST: An immunoglobulin variable domain sequence analysis tool. Nucleic Acids Res..

[36] Gadala-Maria D., Yaari G., Uduman M., Kleinstein S.H. (2015). Automated analysis of high-throughput B-cell sequencing data reveals a high frequency of novel immunoglobulin V gene segment alleles. Proc. Natl. Acad. Sci. USA.

[37] Gupta N.T., Vander Heiden J.A., Uduman M., Gadala-Maria D., Yaari G., Kleinstein S.H. (2015). Change-O: A toolkit for analyzing large-scale B cell immunoglobulin repertoire sequencing data. Bioinformatics.

[38] Hoehn K.B., Pybus O.G., Kleinstein S.H. (2022). Phylogenetic analysis of migration, differentiation, and class switching in B cells. PLoS Comput. Biol..

[39] Lefranc M.P., Giudicelli V., Ginestoux C., Bodmer J., Muller W., Bontrop R., Lemaitre M., Malik A., Barbie V., Chaume D. (1999). IMGT, the international ImMunoGeneTics database. Nucleic Acids Res..

[40] Hoehn K.B., Vander Heiden J.A., Zhou J.Q., Lunter G., Pybus O.G., Kleinstein S.H. (2019). Repertoire-wide phylogenetic models of B cell molecular evolution reveal evolutionary signatures of aging and vaccination. Proc. Natl. Acad. Sci. USA.

[41] Avnir Y., Tallarico A.S., Zhu Q., Bennett A.S., Connelly G., Sheehan J., Sui J., Fahmy A., Huang C.Y., Cadwell G. (2014). Molecular signatures of hemagglutinin stem-directed heterosubtypic human neutralizing antibodies against influenza A viruses. PLoS Pathog..

[42] Joyce M.G., Wheatley A.K., Thomas P.V., Chuang G.Y., Soto C., Bailer R.T., Druz A., Georgiev I.S., Gillespie R.A., Kanekiyo M. (2016). Vaccine-Induced Antibodies that Neutralize Group 1 and Group 2 Influenza A Viruses. Cell.

[43] Wu N.C., Yamayoshi S., Ito M., Uraki R., Kawaoka Y., Wilson I.A. (2018). Recurring and Adaptable Binding Motifs in Broadly Neutralizing Antibodies to Influenza Virus Are Encoded on the D3-9 Segment of the Ig Gene. Cell Host Microbe.

[44] Cheung C.S., Fruehwirth A., Paparoditis P.C.G., Shen C.H., Foglierini M., Joyce M.G., Leung K., Piccoli L., Rawi R., Silacci-Fregni C. (2020). Identification and Structure of a Multidonor Class of Head-Directed Influenza-Neutralizing Antibodies Reveal the Mechanism for Its Recurrent Elicitation. Cell Rep..

[45] Lin T.H., Lee C.D., Fernandez-Quintero M.L., Ferguson J.A., Han J., Zhu X., Yu W., Guthmiller J.J., Krammer F., Wilson P.C. (2025). Structurally convergent antibodies derived from different vaccine strategies target the influenza virus HA anchor epitope with a subset of V(H)3 and V(K)3 genes. Nat. Commun..

[46] Wang Y., Lv H., Teo Q.W., Lei R., Gopal A.B., Ouyang W.O., Yeung Y.H., Tan T.J.C., Choi D., Shen I.R. (2024). An explainable language model for antibody specificity prediction using curated influenza hemagglutinin antibodies. Immunity.

[47] Ende Z., Mishina M., Kauffman R.C., Kumar A., Kumari R., Knight P.R., Sambhara S. (2024). Human monoclonal antibody cloning and expression with overlap extension PCR and short DNA fragments. J. Immunol. Methods.

[48] Jorquera P.A., Mishin V.P., Chesnokov A., Nguyen H.T., Mann B., Garten R., Barnes J., Hodges E., De La Cruz J., Xu X. (2019). Insights into the antigenic advancement of influenza A(H3N2) viruses, 2011–2018. Sci. Rep..

[49] Levine M.Z., Holiday C., Jefferson S., Gross F.L., Liu F., Li S., Friel D., Boutet P., Innis B.L., Mallett C.P. (2019). Heterologous prime-boost with A(H5N1) pandemic influenza vaccines induces broader cross-clade antibody responses than homologous prime-boost. npj Vaccines.

[50] Levine M.Z., Liu F., Bagdasarian N., Holiday C., Jefferson S., Li Z.N., Pappas C., Tumpey T., Uyeki T.M., Mellis A.M. (2025). Neutralizing Antibody Response to Influenza A(H5N1) Virus in Dairy Farm Workers, Michigan, USA. Emerg. Infect. Dis..

[51] WHO Global Influenza Surveillance Network Manual for the Laboratory Diagnosis and Virological Surveillance of Influenza 2011. https://iris.who.int/server/api/core/bitstreams/caddade1-ce40-446e-9267-ddecddcfc943/content.

[52] Kallewaard N.L., Corti D., Collins P.J., Neu U., McAuliffe J.M., Benjamin E., Wachter-Rosati L., Palmer-Hill F.J., Yuan A.Q., Walker P.A. (2016). Structure and Function Analysis of an Antibody Recognizing All Influenza A Subtypes. Cell.

[53] Dreyfus C., Laursen N.S., Kwaks T., Zuijdgeest D., Khayat R., Ekiert D.C., Lee J.H., Metlagel Z., Bujny M.V., Jongeneelen M. (2012). Highly conserved protective epitopes on influenza B viruses. Science.

[54] Ray R., Nait Mohamed F.A., Maurer D.P., Huang J., Alpay B.A., Ronsard L., Xie Z., Han J., Fernandez-Quintero M., Phan Q.A. (2024). Eliciting a single amino acid change by vaccination generates antibody protection against group 1 and group 2 influenza A viruses. Immunity.

[55] Burioni R., Canducci F., Mancini N., Clementi N., Sassi M., De Marco D., Diotti R.A., Saita D., Sampaolo M., Sautto G. (2010). Monoclonal antibodies isolated from human B cells neutralize a broad range of H1 subtype influenza A viruses including swine-origin Influenza virus (S-OIV). Virology.

[56] Chen Y., Wang F., Yin L., Jiang H., Lu X., Bi Y., Zhang W., Shi Y., Burioni R., Tong Z. (2022). Structural basis for a human broadly neutralizing influenza A hemagglutinin stem-specific antibody including H17/18 subtypes. Nat. Commun..

[57] Wu Y., Cho M., Shore D., Song M., Choi J., Jiang T., Deng Y.Q., Bourgeois M., Almli L., Yang H. (2015). A potent broad-spectrum protective human monoclonal antibody crosslinking two haemagglutinin monomers of influenza A virus. Nat. Commun..

[58] DiLillo D.J., Tan G.S., Palese P., Ravetch J.V. (2014). Broadly neutralizing hemagglutinin stalk-specific antibodies require FcgammaR interactions for protection against influenza virus in vivo. Nat. Med..

[59] DiLillo D.J., Palese P., Wilson P.C., Ravetch J.V. (2016). Broadly neutralizing anti-influenza antibodies require Fc receptor engagement for in vivo protection. J. Clin. Investig..

[60] Steventon R., Stolle L., Thompson C.P. (2025). How Broadly Neutralising Antibodies Are Redefining Immunity to Influenza. Antibodies.

[61] Fischer A.A., Corcoran M., Brouwer P.J.M., Chernyshev M., Gillespie R.A., Nicoletto A., Loeffler J.R., Ferguson J.A., Zygouras I., Pushparaj P. (2026). Genetically diverse influenza antibodies highlight the role of IG germline gene variation and inform population-comprehensive vaccine strategies. Immunity.

[62] Daniel K., Ullrich L., Ruchnewitz D., Meijers M., Halwe N.J., Wild U., Eberhardt J., Schon J., Stumpf R., Schlotz M. (2026). Pre-existing neutralizing antibodies against cattle-transmitted influenza A virus H5N1 are detectable in unexposed individuals. Immunity.

