# Supplementary material for: Memory B Cell Repertoire of a Repeatedly Vaccinated Individual Includes Influenza Hemagglutinin-Specific Cross-Group Binders with Affinity Beyond A(H1N1)pdm09 and A(H3N2) Seasonal Strains
Source: Vaccines (Basel). 2026 Aug 27;14(9):742. doi: 10.3390/vaccines14090742 (PMC13611281; doi:10.3390/vaccines14090742)
Supplement: Supplementary file 1 [file vaccines-14-00742-s001.zip › vaccines-4432077 _supplementary Tables.pdf]

Supplementary Tables

Table S1. Recombinant HAs used in the study. All recombinant proteins were expressed in-house in insect cells and obtained commercially (Sino Biological, Paoli, PA, USA) from insect cells or HEK293 mammalian cells as indicated.

Subtype	Source	ID	Strain	Abbreviation	Accession #	Cells	Muta-tion	HA domain	Assay
H1N1	Sino B	11684-V08H2	A/Puerto Rico/8/1934	A/PR/8/1934	ABD77675.1	HEK293	wt	ectodamain/tri-mer	BLI
H1N1pdm9	Sino B	11055-V08H7	A/California/04/2009	A/C/4/2009	ACP41105.1	HEK293	wt	ectodamain/tri-mer	BLI
H1N1pdm9	in house	CDC098	A/California/07/2009		EPI177294	baculo/ in-sect	Y91F	ectodamain/tri-mer	Flow cytometry
H1N1pdm9	in house	CDC078	A/Michigan/45/2015	A/M/45/2015	EPI685579	baculo/ in-sect	wt	ectodamain/tri-mer	BLI
H1N1pdm9	in house	CDC185	A/Michigan/45/2015	Group1HAstem	EPI685579	baculo/ in-sect	wt	HA stem	BLI
H1N1pdm9	in house	CDC243	A/Brisbane/02/2018	A/B/2/2018	EPI1212884	baculo/ in-sect	wt	ectodamain/tri-mer	BLI
H1N1pdm9	in house	CDC331	A/Wisconsin/588/2019	A/W/588/2019	EPI1661758	baculo/ in-sect	wt	ectodamain/tri-mer	BLI
H3N2	in-house	CDC446	A/Hong Kong/1/1968	A/HK/1/1968	AAK51718.1	baculo/ in-sect	Y98F	ectodamain/tri-mer	BLI
H3N2	Sino B	11056-V08B	A/Brisbane/10/2007	A/B/10/2007	ABW23353.1	baculo/ in-sect	wt	Met1-Trp530/monomer	BLI
H3N2	in house	CDC096	A/Texas/50/2012		EPI2318462	baculo/ in-sect	Y98F	ectodamain/tri-mer	Flow cytometry
H3N2	in house	CDC140	A/Singapore/INFIMH-16-0019/2018	A/S/2018	EPI780183	baculo/ in-sect	wt	ectodamain/tri-mer	BLI

H3N2	in house	CDC186	A/Singapore/INFIMH-16-0019/2018	Group2HAstem	EPI780183	baculo/ insect	wt	HA stem	BLI
H3N2	Sino B	40765-V08H2	A/Hong Kong/45/2019	A/HK/45/2019	EPI1588464	HEK293	wt	ectodamain/trimer	BLI
H3N2	in-house	CDC348	A/Cambodia/e0826360/2020	A/C/2020	EPI1837753	baculo/ insect	wt	ectodamain/trimer	BLI
H3N2	Sino B	40859-V08H1	A/Darwin/09/2021	A/D/09/2021	EPI1859996	HEK293	wt	ectodamain/trimer	BLI
H5N1	IRR	FR-86	A/Anhi/01/2005	A/A/1/2005	N/A	baculo/ insect	wt	ectodamain/trimer	BLI
H5N1	IRR	FR-84	A/bar-headed goose/Qinghai /1A/ 2005	A/Q/1A/2005	N/A	baculo/ insect	wt	ectodamain/trimer	BLI
H5N8	IRR	FR-1418	A/gyrfalco/Washington/41088-6/2014	A/W/6/2014	N/A	baculo/ insect	wt	ectodamain/trimer	BLI
H7N9	IRR	FR-69	A/New York/107/2003	A/NY/107/2003	N/A	baculo/ insect	wt	ectodamain/trimer	BLI
H9N2	IRR	FR-88	A/Hong Kong/1073/1999	A/HK/1999	N/A	baculo/ insect	wt	ectodamain/trimer	BLI
H13N9	IRR	FR-73	A/shorebird/Delaware/68/2004	A/D/68/2004	N/A	baculo/ insect	wt	ectodamain/trimer	BLI

Table S2. Seasonal influenza viruses used in the study. All viruses were propagated in-house.

**Subtype/Type**	**Internal ID#**	**Strain**	**Abbreviation**	**Passage history**
A(H1N1)pdm09	2009712112	A/California/07/2009	A/C/7/2009	C4S1(12/03/2024)
A(H1N1)pdm09	3000683658	A/Brisbane/02/2018	A/B/2/2019	E3/E1(09/10/2018)
A(H1N1)pdm09	3026054333	A/Wisconsin/588/2019	A/W/588/2020	C2S1(05/15/2020)
A(H1N1)pdm09	3026053401	A/Hawaii/70/2019	A/H/70/2020	C1S1(04/28/2020)

A(H1N1)pdm09	3030759082	A/Rhode Island/34/2023	A/R I /34/2024	S1(10/25/23) NY
A(H1N1)pdm09	3030759088	A/Georgia/53/2023	A/G/53/2024	S1(10/25/23) NY
A(H3N2)	2009719818	A/Perth/16/2009	A/P/16/2010	EX/S1(07/21/2017)
A(H3N2)	2012784336	A/Victoria/361/2011	A/V/361/2012	C2/C2/C3/C1/S2(12/03/2024)
A(H3N2)	2012704894	A/Texas/50/2012	A/T/50/2013	EX/S2(12/03/2024)
A(H3N2)	2014767086	A/Switzerland/9715293/2013	A/S/2014	EX/S1(08/25/2017)
A(H3N2)	3000095995	A/Hong Kong/4801/2014	A/H K/2015	E5/E2/E2/C1/S1(08/25/2017)
A(H3N2)	3000095146	A/Washington/33/2014	A/W /33/2015	M1/S2(04/27/2020)
A(H3N2)	3025624278	A/Pennsylvania/46/2015	A/P /46/2016	S3(04/27/2020)
A(H3N2)	3000482555	A/Singapore/INFIMH-16-0019/2016	A/S/2016	CXS1(12/03/2024)
A(H3N2)	3025630943	A/Louisiana/50/2017	A/L /50/2018	S2(06/19/2018)
A(H3N2)	3025630942	A/Louisiana/49/2017	A/L /49/2018	S2(06/19/2018)
IBV	3026017997	B/North Carolina/25/2018	B/NC/2019	C2(05/04/2020)
IBV	3026017441	B/Missouri/12/2018	B/M/12/2019	C2(05/04/2020)

Table S3. HA-specific BCR repertoire of memory B cells: list of HA-specific clonotypes. Representative mAbs from multi-cell class-switched clonotypes were tested for binding to A/Michigan/45/15(H1N1)pdm09 and A/Singapore/2016 (H3N2) HA ectodomain trimers as well as Group 1 and Group 2 HA stem [31,32] recombinant proteins (Table S1) during a pilot screening using the method by Ende Z et al. [47]. Three cross-group clonotypes binding to H1-, H3- HAs, and Group 1 and Group 2 HA stems were found: 117_1, 152_44 and 256_4.

Specificity	ID	Rank	Size	VH	DH	JH	CDR3 (H)	V light	J light	CDR3 (light)
**H1/H3/ gr 1/gr 2**	**117_1**	**6**	**99**	**IGHV3-30**	**IGHD6-13**	**IGHJ6**	**CAKVARQQLLFVHSQYYYYYMDVW**	IGKV2-28, IGKV2D-28	**IGKJ5**	**CMQTLQTPITF**
H1	20_0	8	63	IGHV3-48	IGHD4-17	IGHJ3	CAREGGTGDYRDDAFDIW	IGKV1-33, IGKV1D-33	IGKJ3	CQQVRTF
**H1/H3/ gr 1/gr 2**	**152_44**	**9**	**63**	**IGHV1-18**	**None**	**IGHJ4**	**CARGPPQGMVVVGMTENFDFW**	**IGKV3-20**	**IGKJ2**	**CQLYGSSPPRYTF**

H3	45_200	11	41	IGHV1-3	IGHD6-19	IGHJ5	CARIGDSSGFFGMTWNHW	IGLV2-14	IGLJ2, IGLJ3	CSSYTTTSALVLF
H1	737_40	14	31	IGHV1-2	None	IGHJ6	CARGHDILTGFAGGQFFHHMDVW	IGKV2-28, IGKV2D-28	IGKJ1	CMQALQTPAF
H3	131_2	15	31	IGHV4-39	IGHD3-10	IGHJ5	CARQEGDTMVRGLIKDWFDPW	IGLV3-1	IGLJ2, IGLJ3	CQAWDSSTVVF
**H1/H3/ gr 1/gr 2**	**256_4**	**16**	**25**	**IGHV3-30**	**IGHD6-13**	**IGHJ6**	**CAKVARQQQLVFVSTQYYYYYMDVW**	IGKV2-28, IGKV2D-28	**IGKJ5**	**CMQALQTPLTF**
H1	546_33	19	17	IGHV5-51	IGHD5-5, IGHD5-18	IGHJ6	CARQDGGDTAMVGLGYMDVW	IGKV3-15	IGKJ3	CQQYNSWPPESL
H1	713_232	21	16	IGHV1-69	None	IGHJ5	CARASMGIQLWLGTDLW	IGLV2-14	IGLJ2, IGLJ3	CSSYTSTNTVIF
H1	286_173	22	16	IGHV3-23	None	IGHJ6	CAKAANFFYYYMDVW	IGKV3-15	IGKJ4	CQQYNNWPPLTF
H3	689_68	23	15	IGHV1-18	IGHD2-15, IGHD2-2	IGHJ4	CARDCPFSYCSWTSDFW	IGKV3-20	IGKJ4	CQQYGTSPRTF
H1	414_30	26	14	IGHV4-39	IGHD4-17	IGHJ5	CARRLRGDYVGWFDPW	IGLV3-9	IGLJ2, IGLJ3	CQVWDNSAGVVF
H1	288_247	27	14	IGHV1-69	None	IGHJ6	CARGPNYYDSYMDVW	IGKV3-20	IGKJ1	CQQYGSSPRTF
H1	401_266	30	14	IGHV1-69	IGHD3-9	IGHJ4	CAREKGYILTGRFDYW	IGKV3-20	IGKJ2	CQQYGSSPPYTF
H3	689_116	32	12	IGHV1-18	IGHD2-2	IGHJ4	CARDCPFFYCSSTADYW	IGKV3-20	IGKJ5	CQQYGTSPPVTF
H3	720_16	35	11	IGHV4-61	IGHD5-18, IGHD5-12	IGHJ4	CAGKSPNSGFGLCLDHW	IGLV2-14	IGLJ1	CSSYTSSSTLYVF
H3	398_43	36	10	IGHV1-46	None	IGHJ5	CARGHSSAWNWWLDPW	IGKV3-20	IGKJ4	CQQYGRSRLTF
H1	92_96	58	6	IGHV1-69	None	IGHJ4	CARGLSGDYLFRRGLDSW	IGLV2-14	IGLJ2, IGLJ3	CSSYTSSNTLVF

H1	406_63	68	6	IGHV1-69	IGHD3-10	IGHJ4	CARDEYSGSYWGEGDW	IGKV3-11	IGKJ2	CQQRSNWPPLYTF
H3/gr 2	780_571	323	2	IGHV3-30	IGHD3-9	IGHJ6	CAKDHYDIMTGPTYYYYYMDVW	IGLV4-69	IGLJ2, IGLJ3	CQAWGTGVQVVF

Table S4. Comparison of the 117_1 clonotype to previously published bn antibodies with the IGHV3-30 gene. The 5524 immunoglobulin sequences collated in Wang et al. [46] were filtered for those with broadly neutralizing capability and containing the IGHV3-30 gene segment and light chain information. These sequences were compared to 117_1. The 117_1 family shares IGHJ6 and IGKV2-28 usage with only one other clone, 01.e.01_Heavy, which has a different CDR3H length (23 vs 22), shows almost no similarity in CDR3H sequence, and uses different IGKJ genes.

(Supplementary table S4 is provided separately as an Excel file)
