# Supplementary material for: Memory B Cell Repertoire of a Repeatedly Vaccinated Individual Includes Influenza Hemagglutinin-Specific Cross-Group Binders with Affinity Beyond A(H1N1)pdm09 and A(H3N2) Seasonal Strains
Source: Vaccines (Basel). 2026 Aug 27;14(9):742. doi: 10.3390/vaccines14090742 (PMC13611281; doi:10.3390/vaccines14090742)
Supplement: Supplementary file 1 [file vaccines-14-00742-s001.zip › vaccines-4432077_supplementary Figures_20260826.pdf]

## Supplementary Figures

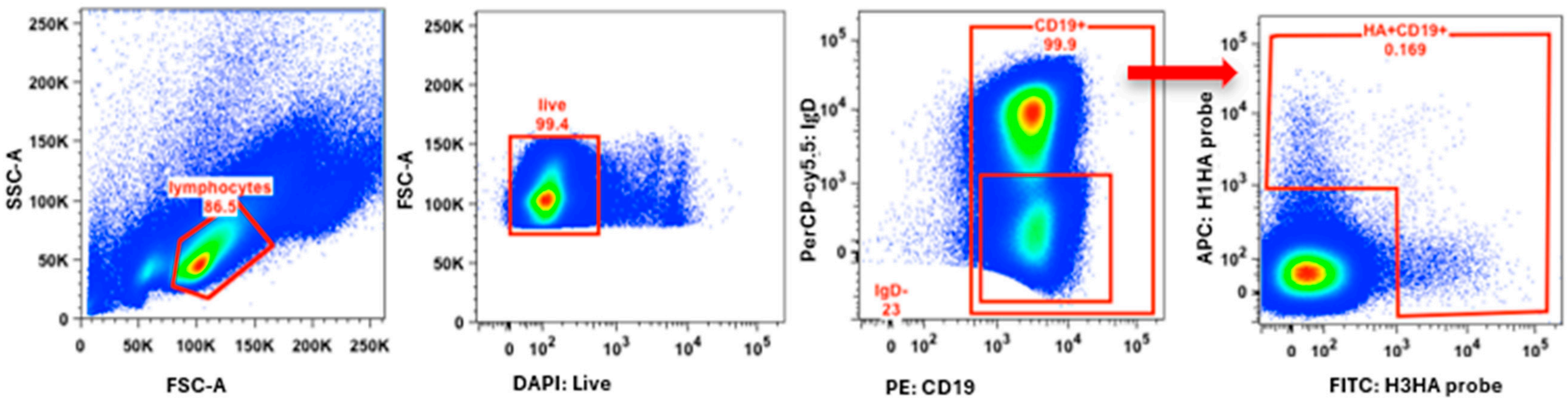


**Figure S1.** Flow cytometry gating for sorting HA-specific B cells. B cells were enriched from stained PBMCs. The single- and double- positive HA probe + CD19+ CD3-CD14-CD16-cells were sorted. Staining panel included A/Texas/50/2012 (H3N2) HA Y98F and A/California/7/2009 (H1N1)pdm09 HA Y91F probes conjugated with DyLight 650 and DyLight 488 (Thermo Fisher), correspondingly (see method section).

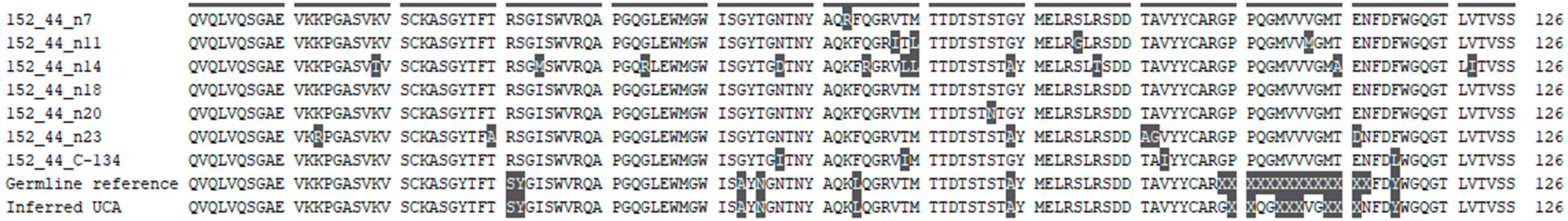
152_44_n7 QVQLVQSGAE VKKPGASVKV SCKASGYTFT RSGISWVRQA PGQGLEWMGW ISGYTGNTNY AQRFQGRVTM TTDTSTSTGY MELRSLRSDD TAVYYCARGP PQGMVVVGMT ENFDFWGQGT LVTVSS 126
152_44_n11 QVQLVQSGAE VKKPGASVKV SCKASGYTFT RSGISWVRQA PGQGLEWMGW ISGYTGNTNY AQKFQGRITL TTDTSTSTGY MELRGLRSDD TAVYYCARGP PQGMVVMGMT ENFDFWGQGT LVTVSS 126
152_44_n14 QVQLVQSGAE VKKPGASVTV SCKASGYTFT RSGMSWVRQA PGQRLEWMGW ISGYTGDTNY AQKFRGRVLL TTDTSTSTAY MELRSLTSDD TAVYYCARGP PQGMVVVGMA ENFDFWGQGT LITVSS 126
152_44_n18 QVQLVQSGAE VKKPGASVKV SCKASGYTFT RSGISWVRQA PGQGLEWMGW ISGYTGNTNY AQKFQGRVTM TTDTSTSTGY MELRSLRSDD TAVYYCARGP PQGMVVVGMT ENFDFWGQGT LVTVSS 126
152_44_n20 QVQLVQSGAE VKKPGASVKV SCKASGYTFT RSGISWVRQA PGQGLEWMGW ISGYTGNTNY AQKFQGRVTM TTDTSTNTGY MELRSLRSDD TAVYYCARGP PQGMVVVGMT ENFDFWGQGT LVTVSS 126
152_44_n23 QVQLVQSGAE VKRPGASVKV SCKASGYTFA RSGISWVRQA PGQGLEWMGW ISGYTGNTNY AQKFQGRVTM TTDTSTSTAY MELRSLRSDD AGVYYCARGP PQGMVVVGMT DNFDFWGQGT LVTVSS 126
152_44_C-134 QVQLVQSGAE VKKPGASVKV SCKASGYTFT RSGISWVRQA PGQGLEWMGW ISGYTGITNY AQKFQGRVIM TTDTSTSTGY MELRSLRSDD TAIYYCARGP PQGMVVVGMT ENFDLWGQGT LVTVSS 126
Germline reference QVQLVQSGAE VKKPGASVKV SCKASGYTFT SYGISWVRQA PGQGLEWMGW ISAYNGNTNY AQKLQGRVTM TTDTSTSTAY MELRSLRSDD TAVYYCARXX XXXXXXXXXX XXFDYWGQGT LVTVSS 126
Inferred UCA QVQLVQSGAE VKKPGASVKV SCKASGYTFT SYGISWVRQA PGQGLEWMGW ISAYNGNTNY AQKLQGRVTM TTDTSTSTAY MELRSLRSDD TAVYYCARGX XQGXXXVGXX XNFDYWGQGT LVTVSS 126

(a)

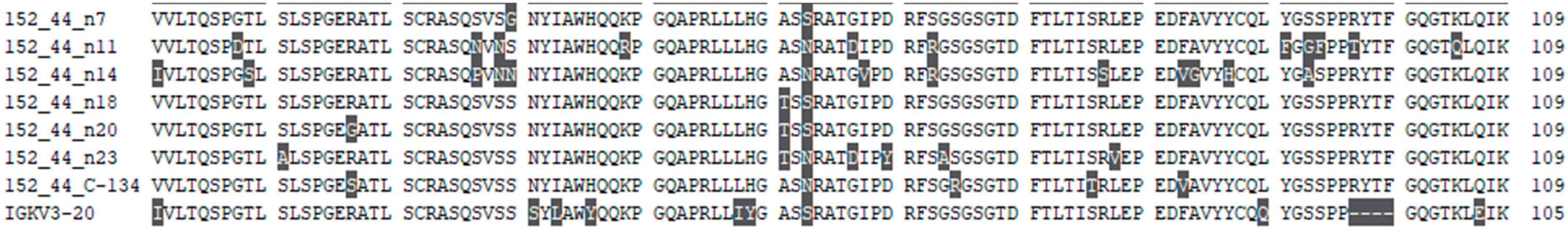
152_44_n7 VVLTQSPGTL SLSPGERATL SCRASQSVSG NYIAWHQQKP GQAPRLLLHG ASSRATGIPD RFSGSGSGTD FTLTISRLEP EDFAVYYCQL YGSSPPRYTF GQGTKLQIK 109
152_44_n11 VVLTQSPDTL SLSPGERATL SCRASQNVNS NYIAWHQQRP GQAPRLLLHG ASNRATDIPD RFRGSGSGTD FTLTISRLEP EDFAVYYCQL FGGFPPTYTF GQGTQLQIK 109
152_44_n14 IVLTQSPGSL SLSPGERATL SCRASQPVNN NYIAWHQQKP GQAPRLLLHG ASNRATGVPD RFRGSGSGTD FTLTISSLEP EDVGVYHCQL YGASPPRYTF GQGTKLQIK 109
152_44_n18 VVLTQSPGTL SLSPGERATL SCRASQSVSS NYIAWHQQKP GQAPRLLLHG TSSRATGIPD RFSGSGSGTD FTLTISRLEP EDFAVYYCQL YGSSPPRYTF GQGTKLQIK 109
152_44_n20 VVLTQSPGTL SLSPGEGATL SCRASQSVSS NYIAWHQQKP GQAPRLLLHG TSSRATGIPD RFSGSGSGTD FTLTISRLEP EDFAVYYCQL YGSSPPRYTF GQGTKLQIK 109
152_44_n23 VVLTQSPGTL ALSPGERATL SCRASQSVSS NYIAWHQQKP GQAPRLLLHG TSNRATDIPY RFSASGSGTD FTLTISRVEP EDFAVYYCQL YGSSPPRYTF GQGTKLQIK 109
152_44_C-134 VVLTQSPGTL SLSPGESATL SCRASQSVSS NYIAWHQQKP GQAPRLLLHG ASNRATGIPD RFSGRGSGTD FTLTITRLEP EDVAVYYCQL YGSSPPRYTF GQGTKLQIK 109
IGKV3-20 IVLTQSPGTL SLSPGERATL SCRASQSVSS SYLAWYQQKP GQAPRLLIYG ASSRATGIPD RFSGSGSGTD FTLTISRLEP EDFAVYYCQQ YGSSPP---- GQGTKLEIK 105

(b)

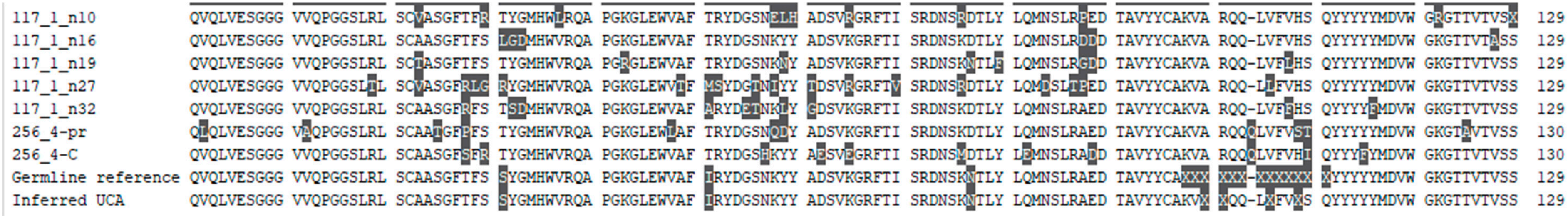
117_1_n10 QVQLVESGGG VVQPGGSLRL SCVASGFTFR TYGMHWLRQA PGKGLEWVAF TRYDGSNELH ADSVRGRFTI SRDNSRDTLY LQMNSLRPED TAVYYCAKVA RQQ-LVFVHS QYYYYYMDVW GRGTTVTVSX 129
117_1_n16 QVQLVESGGG VVQPGGSLRL SCAASGFTFS LGDMHWVRQA PGKGLEWVAF TRYDGSNKYY ADSVKGRFTI SRDNSKDTLY LQMNSLRDDD TAVYYCAKVA RQQ-LVFVHS QYYYYYMDVW GKGTTVTASS 129
117_1_n19 QVQLVESGGG VVQPGGSLRL SCTASGFTFS TYGMHWVRQA PGRGLEWVAF TRYDGSNKNY ADSVKGRFTI SRDNSKNTLF LQMNSLRGDD TAVYYCAKVA RQQ-LVFLHS QYYYYYMDVW GKGTTVTVSS 129
117_1_n27 QVQLVESGGG VVQPGGSLIL SCVASGFRLG RYGMHWVRQA PGKGLEWVIF MSYDGINIYY TDSVRGRFTV SRDNSRDTLY LQMDSLTPED TAVYYCAKVA RQQ-LLFVHS QYYYYYMDVW GKGTTVTVSS 129
117_1_n32 QVQLVESGGG VVQPGGSLRL SCAASGFRFS TSDMHWVRQA PGKGLEWVAF ARYDETNKLY GDSVKGRFTI SRDNSKDTLY LQMNSLRAED TAVYYCAKVA RQQ-LVFEHS QYYYYEMDVW GKGTTVTVSS 129
256_4-pr QLQLVESGGG VAQPGGSLRL SCAATGFPFS TYGMHWVRQA PGKGLEWLAF TRYDGSNQDY ADSVKGRFTI SRDNSKDTLY LQMNSLRAED TAVYYCAKVA RQQQLVFVST QYYYYYMDVW GKGTAVTVSS 130
256_4-C QVQLVESGGG VVQPGGSLRL SCAASGFSFR TYGMHWVRQA PGKGLEWVAF TRYDGSHKYY AESVEGRFTI SRDNSMDTLY LEMNSLRADD TAVYYCAKVA RQQQLVFVHI QYYYFYMDVW GKGTTVTVSS 130
Germline reference QVQLVESGGG VVQPGGSLRL SCAASGFTFS SYGMHWVRQA PGKGLEWVAF IRYDGSNKYY ADSVKGRFTI SRDNSKNTLY LQMNSLRAED TAVYYCAXXX XXX-XXXXXX XYYYYYMDVW GKGTTVTVSS 129
Inferred UCA QVQLVESGGG VVQPGGSLRL SCAASGFTFS SYGMHWVRQA PGKGLEWVAF IRYDGSNKYY ADSVKGRFTI SRDNSKNTLY LQMNSLRAED TAVYYCAKVX XQQ-LXFVXS QYYYYYMDVW GKGTTVTVSS 129

(c)

```
117_1_n 10_kappa -IVLTQSPVS LPVTPGEPAS ISCRSSQSLL HGNGYTYLEW YLQKPGQSPQ LLIYLGSNRA SGVPDRFSGS GSGTDFTLKI STVEAEDVGL YYCMQALQTP ITFGQGTRLQ IK  111
117_1_n 16_kappa DIVMTQSPLS LPVTPGEPAS ISCRSSQSLL HGNGYKYLDW YLQKPGQSPQ LLIYLGSNRA SGVPDRFSGS GSGTDFTLKI SRVEAEDVGV YYCMQTLQTP ITFGQGTRLE IK  112
117_1_n19_kappa  DIVMTQSPLS LPVTPGEPAS ISCRSSQSLL HGNGDNYLNW YLRKPGQSPQ LLIYLGSNRA SGVPDRFSGS GSGTDFTLKI SRVEAEDVGV YYCMQALQIP ITFGQGTRLE IK  112
117_1_n27_kappa  DIVLTQSPLS LPVTPGETAS ISCRSSQSLL HGNGNNYLEW YLQKPGQSPQ LLIYLGSNRA SGVPDRFSGS GSGTDFTLKI SSMEAEDVGV YYCMQTLQTP ITFGQGTRVE TK  112
117_1_n32_kappa  DILMTQSPLS LPVTPGEPAS ISCRSSQSLL HGNGDNYLDW YLQKPGQSPQ VLIYLGSNRA SGVPDRFSGS GSGTDFTLKI SRVEAEDVGV YYCMQALQIP ITFGQGTRLE IK  112
256_4-pr_kappa   DIVLTQSPLS LPVTPGEPAS ISCRSSQSLL RGNGYNYLHW YLQKPGQSPQ LLIYLGSNRA SGVPDRFSGS GSGTYFTLKI SRVEADDVGI YSCMQALQTP LTFGQGTRLD IK  112
256_4-C_kappa    -IVMTQSPLS LPVTPGEPAS ISCRSSQSLL HGNGYNYLDW YLQKPGQSPQ LLIYLGSNRA SGVPDRFSGS GSGTDFTLKI SRVEADDVGI YYCMQSLQTP ITFGQGTRLE SK  111
IGKV2-28         DIVMTQSPLS LPVTPGEPAS ISCRSSQSLL HSNGYNYLDW YLQKPGQSPQ LLIYLGSNRA SGVPDRFSGS GSGTDFTLKI SRVEAEDVGV YYCMQALQTP ---GQGTRLE IK  109
```

**(d)**

**Figure S2.** The representative mAbs from the cross-reactive clonotypes were expressed (Sino Biological, Paoli, PA, USA) for additional testing after the pilot screening of supernatants from mAb construct transfection. Protein sequence alignment for 152_44 heavy (a) and light (b) chains and 117_1/256_4 heavy (c) and light (d) chains. The clonotypes 117_1 and 256_4 shared the same molecular signature: same germline genes, highly similar CDR3 for heavy and light chains but 1 amino acid difference in the length of CDRH3; and considered as a cluster (117_1/256_4) for testing affinity and neutralization. The alignment was done with Geneious Prime. The alignments included inferred UCAs.

(a)

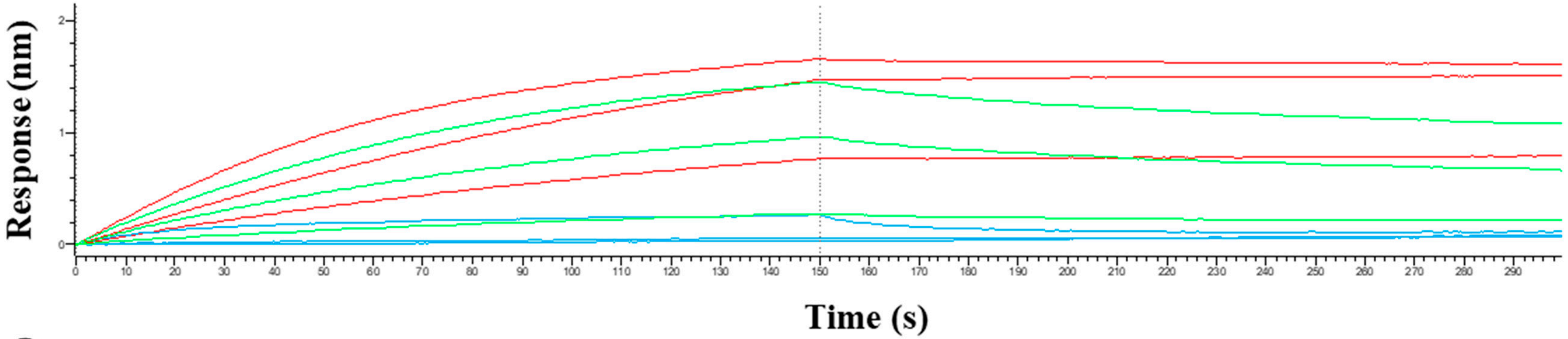
Response (nm)
2
1
0
0 10 20 30 40 50 60 70 80 90 100 110 120 130 140 150 160 170 180 190 200 210 220 230 240 250 260 270 280 290
Time (s)

(b)

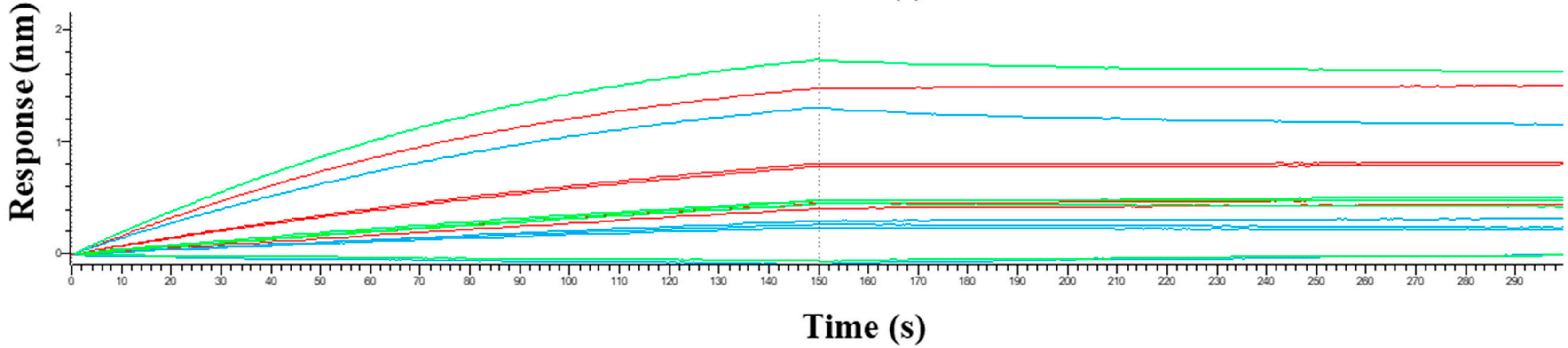
Response (nm)
2
1
0
0 10 20 30 40 50 60 70 80 90 100 110 120 130 140 150 160 170 180 190 200 210 220 230 240 250 260 270 280 290
Time (s)

(c)

| HA (IAV strain) | Subtype | mAb | Response, nm |
|---|---|---|---|
| A/California/04/2009 | A(H1N1)pdm09 | UCA_154-22 | 0.26 |
| A/California/04/2009 | A(H1N1)pdm09 | UCA_154-22_N55T | 1.43 |
| A/California/04/2009 | A(H1N1)pdm09 | 154_44_n11 | 1.63 |
| A/Michigan/45/2015 | A(H1N1)pdm09 | UCA_154-22 | 0.06 |
| A/Michigan/45/2015 | A(H1N1)pdm09 | UCA_154-22_N55T | 0.94 |
| A/Michigan/45/2015 | A(H1N1)pdm09 | 154_44_n11 | 1.43 |
| A/Wisconsin/588/2019 | A(H1N1)pdm09 | UCA_154-22 | 0.03 |
| A/Wisconsin/588/2019 | A(H1N1)pdm09 | UCA_154-22_N55T | 0.27 |
| A/Wisconsin/588/2019 | A(H1N1)pdm09 | 154_44_n11 | 0.75 |
| A/Brisbane/10/2007 | A(H3N2) | UCA_154-22 | -0.09 |
| A/Brisbane/10/2007 | A(H3N2) | UCA_154-22_N55T | -0.06 |
| A/Brisbane/10/2007 | A(H3N2) | 154_44_n11 | 0.39 |
| A/Cambodia/e0826360/2020 | A(H3N2) | UCA_154-22 | 0.28 |
| A/Cambodia/e0826360/2020 | A(H3N2) | UCA_154-22_N55T | 0.45 |
| A/Cambodia/e0826360/2020 | A(H3N2) | 154_44_n11 | 0.75 |
| A/Darwin/09/2021 | A(H3N2) | UCA_154-22 | 1.28 |
| A/Darwin/09/2021 | A(H3N2) | UCA_154-22_N55T | 1.70 |
| A/Darwin/09/2021 | A(H3N2) | 154_44_n11 | 1.45 |
| A/Hong Kong/45/2019 | A(H3N2) | UCA_154-22 | 0.23 |
| A/Hong Kong/45/2019 | A(H3N2) | UCA_154-22_N55T | 0.46 |
| A/Hong Kong/45/2019 | A(H3N2) | 154_44_n11 | 0.43 |
| A/Singapore/INFIMH-16-0019/2018 | A(H3N2) | UCA_154-22 | 0.25 |
| A/Singapore/INFIMH-16-0019/2018 | A(H3N2) | UCA_154-22_N55T | 0.42 |
| A/Singapore/INFIMH-16-0019/2018 | A(H3N2) | 154_44_n11 | 0.78 |

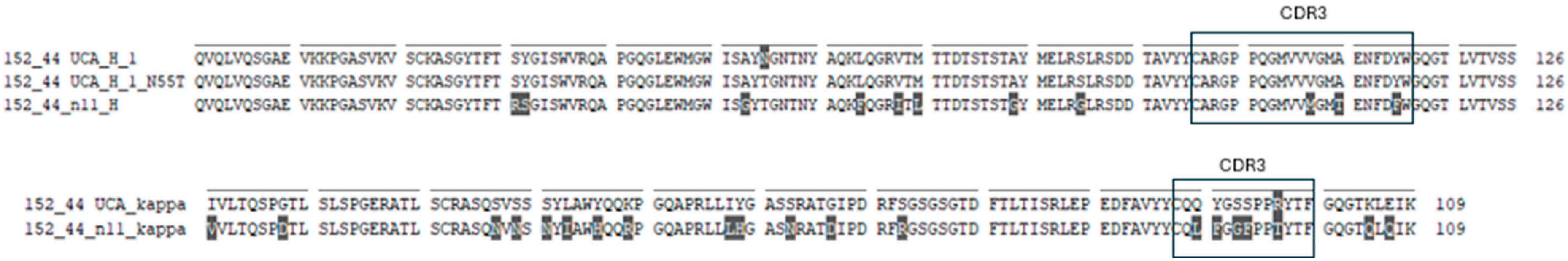


**(d)**

**Figure S3.** Binding of 152_44 UCA and UCA_N55T mAbs to A(H1) and A(H3) recHAs. BLI traces for mAb binding to A(H1) (a) and A(H3) (b) recHAs (Table S1) and the corresponding BLI responses (c). VH and VK protein alignment of the tested mAbs was done with Geneious Prime (d). The following color-coding is used: Unmutated Common Ancestor (UCA) in blue, UCA_N55T in green, Node (representative mAb 152_44 n11) in red.

**(a)**

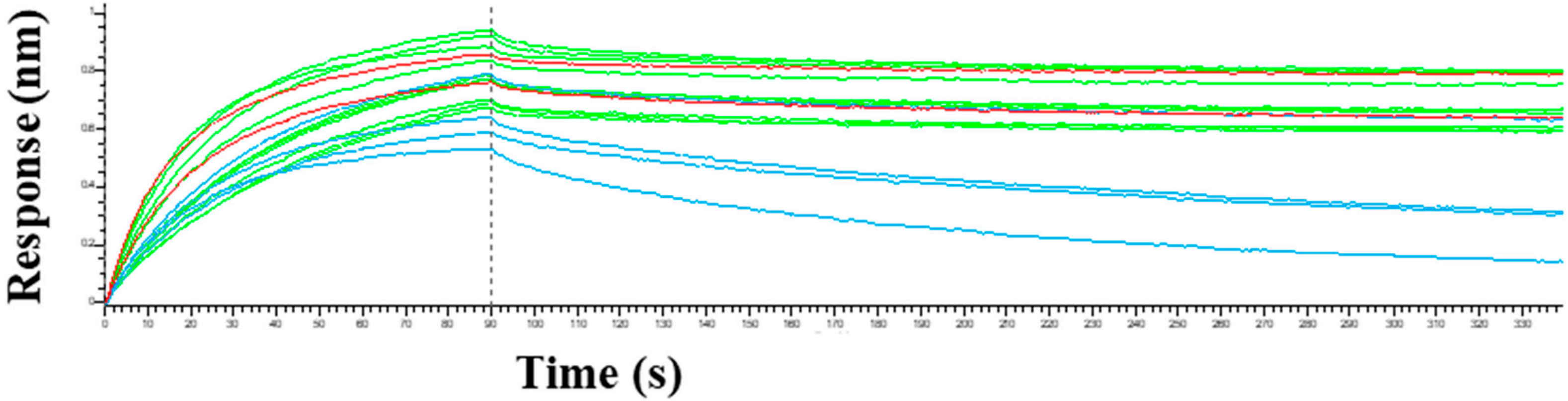


**(b)**

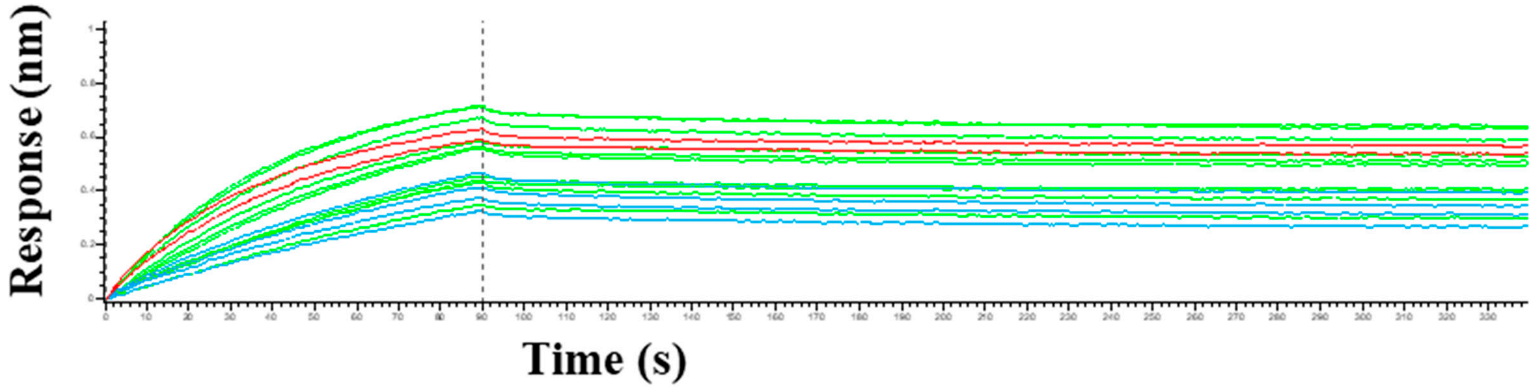

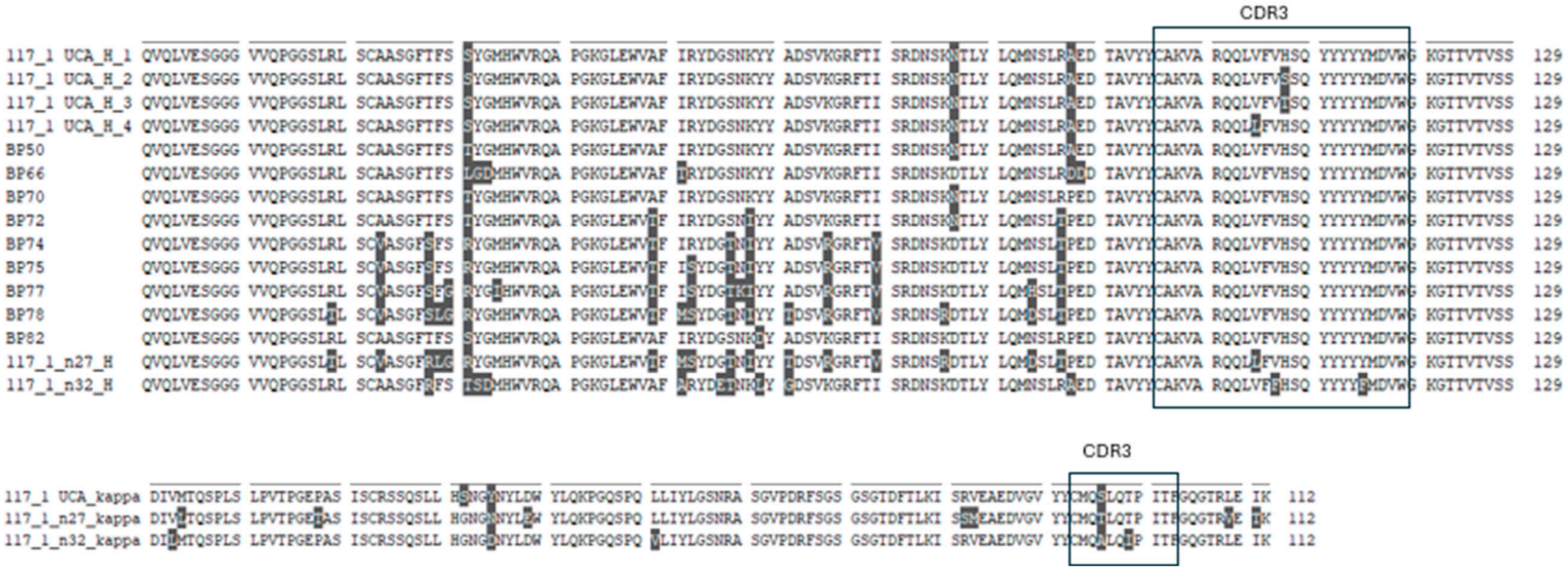


**(c)**

**Figure S4.** Binding of 117_1 UCA and BP mABs to A(H1) and A(H3) recHAs. BLI traces of mAbs binding to A/Brisbane/02/2018 (H1N1)pdm09 (a) and A/Singapore/INFIMH-16-0019/2016(H3N2) (b) recHAs. VH and VK protein alignment for mAbs (UCAs, BPs and representative nodes) were performed using Geneious Prime software (c). All UCAs and BPs shared the same light chain constructed as germline sequences with CDR3 from 117_1 n1 (see method section). The following color-coding is used: Unmutated Common Ancestor (UCA) in blue, Branch point (BP) in green, Node in red.
